# Retinal electrophysiology in central nervous system disorders. A review of human and mouse studies

**DOI:** 10.3389/fnins.2023.1215097

**Published:** 2023-08-02

**Authors:** Paul A. Constable, Jeremiah K. H. Lim, Dorothy A. Thompson

**Affiliations:** ^1^College of Nursing and Health Sciences, Caring Futures Institute, Flinders University, Adelaide, SA, Australia; ^2^Discipline of Optometry, School of Allied Health, University of Western Australia, Perth, WA, Australia; ^3^The Tony Kriss Visual Electrophysiology Unit, Clinical and Academic Department of Ophthalmology, Great Ormond Street Hospital for Children NHS Trust, London, United Kingdom; ^4^UCL Great Ormond Street Institute of Child Health, University College London, London, United Kingdom

**Keywords:** psychiatry, retinal biomarkers, retina, PERG, electro-oculogram, electroretinogram, central nervous system, ERG

## Abstract

The retina and brain share similar neurochemistry and neurodevelopmental origins, with the retina, often viewed as a “window to the brain.” With retinal measures of structure and function becoming easier to obtain in clinical populations there is a growing interest in using retinal findings as potential biomarkers for disorders affecting the central nervous system. Functional retinal biomarkers, such as the electroretinogram, show promise in neurological disorders, despite having limitations imposed by the existence of overlapping genetic markers, clinical traits or the effects of medications that may reduce their specificity in some conditions. This narrative review summarizes the principal functional retinal findings in central nervous system disorders and related mouse models and provides a background to the main excitatory and inhibitory retinal neurotransmitters that have been implicated to explain the visual electrophysiological findings. These changes in retinal neurochemistry may contribute to our understanding of these conditions based on the findings of retinal electrophysiological tests such as the flash, pattern, multifocal electroretinograms, and electro-oculogram. It is likely that future applications of signal analysis and machine learning algorithms will offer new insights into the pathophysiology, classification, and progression of these clinical disorders including autism, attention deficit/hyperactivity disorder, bipolar disorder, schizophrenia, depression, Parkinson’s, and Alzheimer’s disease. New clinical applications of visual electrophysiology to this field may lead to earlier, more accurate diagnoses and better targeted therapeutic interventions benefiting individual patients and clinicians managing these individuals and their families.

## Background

Several reviews and commentaries covering the findings and applications of visual electrophysiology in central nervous system (CNS) disorders have been published in the last decade advocating the retina as a “window to the brain” (London et al., 2013; Lavoie et al., 2014b,c; Gagné et al., 2015; Nguyen et al., 2017; Youssef et al., 2019; Silverstein and Thompson, 2020; Diamond et al., 2022; Silverstein et al., 2022; Schwitzer et al., 2022b,c; Alves et al., 2023; Mahroo, 2023; Tursini et al., 2023) and on using the electroretinogram (ERG) to differentiate schizophrenia and bipolar disorder (Lavoie et al., 2014b; Hébert et al., 2015, 2020; Hosak et al., 2018; Almonte et al., 2020; Peredo et al., 2020, 2022; Silverstein et al., 2020, 2022; Maziade et al., 2022; Moreau et al., 2022; Tursini et al., 2022). To understand the landscape of potential retinal biomarkers in CNS disorders, here we review studies encompassing schizophrenia, bipolar disorder, autism spectrum disorder (ASD), attention deficit hyperactivity disorder (ADHD), seasonal affective disorder, major depressive disorder, Parkinson’s disease (PD) and Alzheimer’s disease (AD). We aim to provide an overview of this developing field with an emphasis on the functional retinal electrophysiological findings. In addition, we have reviewed electrophysiological findings from mouse models of these human CNS based disorders. The reader is referred to Komatsu et al. (2022) for a review on structural findings in this field and Loo et al. (2016) for a review on cortical electrophysiological findings.

### Neurotransmitters in retinal circuits

The main neurotransmitters shared by the cortex and retina are glutamate, dopamine, glycine, and Gamma-Aminobutyric Acid (GABA) which have all been linked to the pathophysiology of neurological disorders (Yang, 2004; Zhou and Danbolt, 2013; Lavoie et al., 2014b; Naaijen et al., 2017; Nguyen et al., 2017; Boccuni and Fairless, 2022). One issue that hinders the direct association of neurological conditions with an electrophysiological biomarker is the overlap between genetic mutations and the clinical phenotypes (Knott et al., 2021; Cabana-Domínguez et al., 2022; Krakowski et al., 2022). For example, ASD, ADHD, and schizophrenia all share common variants in genes encoding GABA and glutamate signaling pathways (Kenny et al., 2014; Chisholm et al., 2015; Fatemi and Folsom, 2015; Naaijen et al., 2017). Furthermore, serotonin and dopamine pathways, that modulate mood (Ruhé et al., 2007), are also associated with many conditions, such as Obsessive–Compulsive Disorder, ADHD, ASD, schizophrenia, and bipolar disorders, and may contribute to anxiety and depression in these conditions (Simonoff et al., 2008; Pagan et al., 2014; Lavoie et al., 2014b; Cabana-Domínguez et al., 2022). Several early studies identified the potential benefit of investigating retinal markers in neurological conditions such as schizophrenia (Raese et al., 1982), ASD (Ritvo et al., 1988; Realmuto et al., 1989) and PD (Terziivanov et al., 1983; Nightingale et al., 1986; Gottlob et al., 1987; Jaffe et al., 1987; Castrogiovanni and Marazziti, 1989; Calzetti et al., 1990). The recent expansion and international standardization of clinical protocols (Bach et al., 2013; Constable et al., 2017; Frishman et al., 2018; Sustar et al., 2018; Thompson et al., 2018; Johnson et al., 2019; Perlman et al., 2020; Hoffmann et al., 2021; Robson et al., 2022; McCulloch et al., 2023), coupled with the relative ease of recording and analyzing the ERG with modern equipment has stimulated interest in using the ERG waveform to better characterize neurological disorders (Mahroo, 2023).

The potential of using the ERG waveform, as a biomarker for CNS based neurological conditions is the goal. The ERG offers a relatively easy and direct measurement of the retina, a neural tissue which shares neurotransmitters in common with the brain. The same neurotransmitters are implicated in the pathogenesis of developmental and degenerative CNS based conditions (London et al., 2013; Hamilton, 2021; Silverstein et al., 2022; Tursini et al., 2022; Alves et al., 2023; Mahroo, 2023). It is likely that a combination of measurements will strengthen the clinical utility of visual electrophysiology in the quest for biomarkers of psychiatric conditions. This will provide permutations of features that may classify and monitor progression of these conditions, such as from retinal and cortical responses (Tursini et al., 2023), or imaging the individual layers of the retina using optical coherence tomography (OCT) to provide an association of structure and functional change within the retina (Murthy et al., 2016).

### The electroretinogram

The ERG waveform is shaped by several neural generators including photoreceptors, bipolar, horizontal, ganglion, amacrine and glial cells whose signaling may be altered in disorders affecting the CNS (Teleanu et al., 2022). The relative contribution of excitatory and inhibitory neurotransmitters gives rise to the amplitude and timing of the ERG’s principal peaks (Robson et al., 2022). Thus, the ERG waveform and its analysis provides a “window” into the brain owing to its well characterized and layered neuronal circuits that share the same principal neurotransmitters as the brain (London et al., 2013).

The specific neural circuits that contribute to the ERG waveform depend upon the state of retinal light adaptation as well as the color, frequency, and strength of the stimulating flash of light that initiates the electrical response of the retina (Robson et al., 2022). In response to a flash of light the initial negative a-wave is derived mainly from the hyperpolarization of photoreceptors due to the phototransduction cascade (Chen, 2005). Hyperpolarization of rods under dark-adapted (DA) and cones under light-adapted (LA) conditions are the main contributors to the amplitude and timing of the a-wave (Baylor et al., 1979; Robson et al., 2003; Robson and Frishman, 2014). The LA a-wave is also shaped by post-receptoral neurons in the inner retina that relate to the OFF signaling pathway (Bush and Sieving, 1994; Gouras and MacKay, 2000; Dang et al., 2011). In addition, horizontal cells provide feedback inhibition to photoreceptors and feedforward inhibition to bipolar cells using GABA signaling pathways (Diamond, 2017; Hirano et al., 2020). The b-wave which is the positive peak following the a-wave is generated by the depolarization of the bipolar cells when glutamate release from the hyperpolarized photoreceptors is reduced following phototransduction of light. ON-pathway bipolar cells have metabotropic glutamate receptors and OFF-pathway bipolar cells have ionotropic glutamate receptors (Knapp and Schiller, 1984; Heynen and van Norren, 1985; Hanna and Calkins, 2006, 2007; Boccuni and Fairless, 2022). In addition, glial cell potassium currents from Müller cells also shape the b-wave (Thompson et al., 2011) and GABAergic inhibitory horizontal cells also contribute to the amplitude of the b-wave (Diamond, 2017; Barnes et al., 2020).

There are a series of small oscillations visible on the ascending limb of the b-wave, termed the oscillatory potentials (OPs) that derive from spiking amacrine cells that use dopamine, GABA, and glycine as their main neurotransmitters (Wachtmeister and Dowling, 1978; Wachtmeister, 1980, 1981, 1998; Contini and Raviola, 2003; Diamond, 2017). The descending limb of the b-wave is shaped by contribution from the retinal ganglion cells (RGCs; Viswanathan et al., 2001) and is termed the photopic negative response (PhNR; Frishman et al., 2018). An alternative way of eliciting the RGC response is to use a very dim light source after an extended period of dark adaptation. The recorded electrical response is known as the scotopic threshold response (STR), which comprises an initial positive component believed to originate from the RGCs and a subsequent negative component thought to receive input from both ganglion and amacrine cells based on mouse studies (Saszik et al., 2002; Bui and Fortune, 2004).

### Other retinal electrophysiological tests

Additional tests include the clinical electro-oculogram (EOG) which measures rod and retinal pigment epithelial (RPE) cell interactions (Constable et al., 2017). In response to light, the basolateral membrane of the RPE depolarizes following a series of intracellular calcium signaling pathways that depend upon bestrophin, which is a calcium and chloride ion regulator (Arden and Constable, 2006; Constable et al., 2006; Constable, 2011, 2014; Cordeiro and Strauss, 2011; Johnson et al., 2017; Constable and Ngo, 2018; Cordes et al., 2020; Constable and Kapoor, 2021). The full field flash ERG and EOG are measures of global retinal activity summated across the whole retinal area. A localized area of central retinal can be assessed using the pattern ERG (PERG; Bach et al., 2013). The PERG is driven by macular cones but dominated by RGC function responding to an alternating chequerboard pattern display that elicits a transient or at higher pattern reversal frequencies, a steady state (ss) response. The multifocal ERG (mfERG; Hoffmann et al., 2021) provides measures of even smaller, geographically localized areas of retinal cone function. Other, extended ERG protocols have been developed to explore specific aspects of the retina. These include the measurement of DA cones (the x-wave) detected when a DA ERG is recorded using a red flash stimulus (Thompson et al., 2018). The extended flash duration under LA conditions which separates the contributions of the ON-and OFF-pathways (Sustar et al., 2018) and a specific S-cone response which is observed using a blue stimulus on an orange background (Perlman et al., 2020).

**Figure 1:**
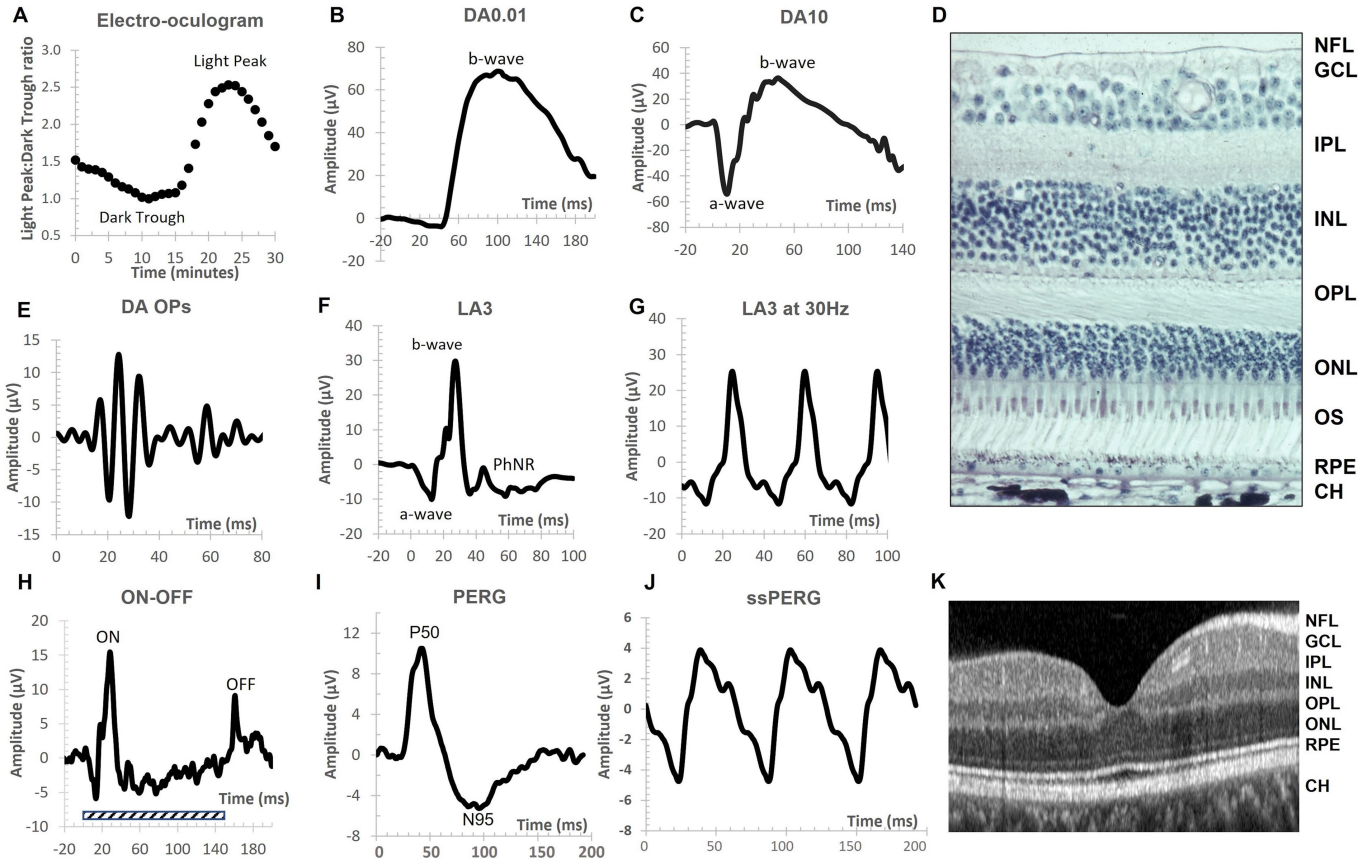
The main electrophysiological measures used to assess disorders of the central nervous system (CNS) reported in this review. Conventional nomenclature for an electroretinogram (ERG) is to name it by the state of retinal adaptation [either dark adapted (DA) or light adapted (LA)] and the flash strength in cd.s.m^−2^. Thus, the DA0.01 is the ERG of the DA retina to a 0.01 cd.s.m^−2^ flash strength. Note the different amplitude and time scales for each representative recording. **(A)** The electro-oculogram (EOG) is a slow response. The dark trough develops during 15 min DA before the onset of constant light causes the basolateral membrane of the retinal pigment epithelium (RPE) to depolarize and reach a maximum voltage difference around 8–10 min following a series of intracellular signaling pathways linked to calcium and bestrophin. The ratio of the light peak to the dark trough voltage is an index of RPE and rod photoreceptor function with recordings taken at 1-min intervals in the dark and light phases. **(B)** The DA0.01 ERG b-wave is the depolarizing response of the rod ON-bipolar cells when metabotropic glutamate receptors signal a fall in glutamate release from the rod outer segments. **(C)** The DA10 waveform has a prominent a-wave derived mainly from hyperpolarization of rods with some cone contribution. The b-wave is derived mainly from depolarization of bipolar cells and shaped by some potassium currents in the Müller cells. Note the DA10 better defines a single a-wave trough compared to the DA3 (not shown). **(D)** Transverse histological section of human retina showing the layered structure of the rod and cone photoreceptor (PR) layer that interdigitate with the supporting RPE layer with vascular choroid (CH) below. The nuclei of the PRs are found in the outer nuclear layer (ONL). The outer plexiform layer (OPL) consists of synapses between ON-, OFF-, and rod bipolar cells with PRs and horizontal cells. The inner nuclear layer contains the nuclei of bipolar, amacrine, horizontal and the Müller glial cells. The cells in the ONL and INL contribute to the a-wave, b-wave, and oscillatory potentials (OPs) of the ERG. The inner plexiform layer (IPL) contains synapses between, bipolar, amacrine and ganglion cells. The ganglion cell layer (GCL) contains the nuclei of retinal ganglion cells whose axons form the nerve fiber layer (NFL) and convey visual information to the lateral geniculate nucleus. **(E)** The OPs are the high frequency components observed in the ascending limb of the DA or LA b-wave and are initiated by the amacrine cells. The OPs waveform is extracted through band pass filtering the original ERG waveform between 100 and 300 Hz. The OPs typically form 5 peaks with the second (OP2) the largest of the series. **(F)** The LA3 a-wave derives from the hyperpolarization of cone outer segments with feedback from OFF-pathway neurons. The b-wave is shaped by ON-and OFF- bipolar cells with amacrine cells contributing to the LA OPs visible on the ascending limb of the b-wave. The descending limb is shaped in part by ganglion cells that form the Photopic Negative Response (PhNR) a minimum that occurs typically at 72 ms after stimulus onset. **(G)** The 30 Hz flicker is a LA3 cd.s.m^−2^ strength presented at 30 Hz. This temporal rate is so high that the ERG waveform becomes sinusoidal shaped. It is driven by long and mid wavelength cones. **(H)** The extended ON/OFF waveform is performed under LA conditions with an extended flash duration indicated by the shaded rectangle. At stimulus onset the b-wave component of the ON-pathway is visible and at the end of the flash the b-wave of the OFF-pathway is visible (termed the d-wave). **(I)** The transient pattern ERG (PERG) is the response of the central macular region of the retina (typically a 15- or 30-degree field) using equal numbers of high contrast black and white cheques that alternate so that stimulus luminance is constant. The main PERG responses are the positive peak at approximately 50 ms (P50) and the negative trough at approximately 95 ms (N95). The N95 is defined by ganglion cell activity whilst the P50 is driven by macular cones but is a mixed response of ganglion cells and second order neurons within the retina. **(J)** The steady state PERG (ssPERG) is recorded with a high reversal rate so that there is a superimposition of the P50 forming a sinusoidal waveform shape. **(K)** An Optical Coherence Tomography (OCT) scan of human fovea shows the layered structure of the retina with the NFL, GCL, IPL, INL, OPL, ONL, RPE and CH visible. The layered structure of the retina, combined with knowledge of the cellular origins and their neurotransmitters of the electrophysiological waveforms has enhanced the application of visual electrophysiology in central nervous system disorders.

Figure 1 provides an overview of the different waveforms that can be recorded under DA and LA conditions to assess primarily the rod, cone and RGCs. For further information on clinical electrophysiological waveforms see Robson et al. (2018).

### Analysis of the waveforms

Although direct measures of amplitude, peak time or amplitude ratios are used as clinically significant markers of the ERG, PERG, mfERG, and EOG, additional mathematical modelling and analyses can provide further detailed insights into the underlying cellular mechanisms and potential contribution to the pathophysiology of CNS disorders. The a-wave has been modelled extensively based on the kinetics of the phototransduction cascade to evaluate this biochemical pathway and gating of ion channels in the outer segments of the photoreceptors (Smith and Lamb, 1997; Friedburg et al., 2004; Mahroo et al., 2012). The luminance response function, which typically models the changes in the b-wave amplitude to a series of flash strengths under DA (Johnson et al., 2019) and LA (Rufiange et al., 2003) conditions has been used to assess retinal sensitivity and the contributions of the cone ON-and OFF-pathways (Hamilton et al., 2007). The OPs which are the high frequency components of the ERG that are extracted from the b-wave signal, can be analyzed in several ways, including calculating the summated amplitudes and timings of each OP or using the integrated root-mean-square of their amplitudes (Robson et al., 2022) or by signal analysis (Forte et al., 2008; Gauvin et al., 2016).

Signal analysis of the ERG waveform can provide an extra layer of resolution in the time-frequency domain that may complement time-domain analysis techniques of amplitude and peak time of the main components of the ERG waveform. Gauvin and colleagues have provided a comprehensive deconstruction of the ERG waveform using discrete wavelet transform analysis. This provides insights into the contribution of the energy produced within the ON-and OFF-pathways localized not only to the a- and b-waves but also to the early and later OPs (Gauvin et al., 2014, 2015, 2017). The application of Gauvin’s work has been instrumental in the use of wavelet analyses and other signal analytical methods in the mfERG and ERG in retinal and neurodevelopmental disorders to date in animal and human studies (Brandao et al., 2017; Dorfman et al., 2020; Constable et al., 2022; Mohammad-Manjur et al., 2022; Sarossy et al., 2022; Zhdanov et al., 2023).

Given the overlapping nature of clinical and genetic features associated with CNS disorders, for this narrative review we have organized the conditions based on the typical age of onset/diagnosis. Thus, ASD and ADHD are considered childhood onset, schizophrenia, major depressive disorder, seasonal affective disorder and bipolar disorder as early adulthood onset and Parkinson’s Disease and Alzheimer’s Disease as late adult onset. First, we present the main human studies, followed by examples from the mouse models of these conditions which have contributed to our understanding of the clinical human findings. The discussion summarizes the main results with respect to neural pathways in the retina associated with the conditions and suggests areas of future study. It should be noted that ERGs performed prior to the first International Society for Clinical Electrophysiology of Vision (ISCEV) standard (Marmor et al., 1989) used protocols with different properties to examine the rod and cone responses under DA and LA conditions. In addition, the Diagnostic and Statistical Manual (DSM) and International Classification of Diseases (ICD) diagnostic classifications may vary in each revision and the diagnoses in each paper reflect the diagnostic and classification descriptions at the time of the study.

### Search method

Table 1 summarizes the main classification codes according to the DSM-5 (American Psychiatric Association, 2013) and the World Health Organization ICD-11 (World Health Organization, 2019) for the conditions included in this review. We excluded studies relating to substance abuse such as cocaine or marijuana and eating disorders. The main electrophysiological measures covered are the ERG, PERG (transient and steady state), mfERG, and the EOG with cortical responses [visual evoked potentials (VEPs)] only discussed where relevant to the study.

**Table 1:** Summary of conditions in which visual electrophysiological studies have been performed based on DSM-V and ICD-11 classification codes.

Condition	DSM-V	ICD-11
Autism spectrum disorder	299.00	6A02.0
Attention deficit hyperactivity disorder	314.00	6A05.2
Attention deficit hyperactivity disorder-inattentive subtype (ADD)	314.01	6A05.0
Schizophrenia	295.90	6A25.0
Bipolar affected disorder	296.00	6A60.0
Major depressive disorder	296.20	6A70.3
Seasonal affective disorder	296.22	6A71.Z
Parkinson’s disease	332.00	8A00.0
Alzheimer’s disease	331.00	8A20

References for this narrative review were selected by searching PubMed for full-text articles and abstracts in English, including mouse and human from 1985 excluding case-reports, using various combinations of the key terms: “electroretinogram,” “multifocal electroretinogram,” “pattern electroretinogram,” “electro-oculogram,” “light-rise,” “a-wave,” “b-wave,” “photopic negative response,” “autism,” “Alzheimer’s,” “schizophrenia,” “bipolar disorder,” “depression,” “Parkinson’s,” “major depressive disorder,” “seasonal affective disorder,” and “dementia.” Additional relevant papers were identified from the reference lists of the selected articles and a final review of PubMed on June 20^th^, 2023, to identify any new publications in this field.

## Results

### Childhood onset

This section provides a summary of the main retinal findings in conditions that are typically diagnosed in childhood. We acknowledge that ASD and ADHD may be diagnosed in adulthood and is a lifelong condition affecting the individual’s quality of life. Table 2 indicates the main reported findings from the studies to date. The abbreviations, “nt” refers to the test not performed, i.e., not tested, “nr” refers to a test described in the methods, but the main results not reported or discussed.

**Table 2:** Summary of findings in studies reported in this review.

	Time to Peak		Amplitude					RGCs	
Study	a-wave	b-wave	LRF	a-wave	b-wave	OPs	Flicker	PhNR	PERG
Autism spectrum disorder									
Ritvo et al. (1988)	LA nr	LA nr	nt	LA nr	DA ↓ LA nr	nt	DA nr	nt	nt
Constable et al. (2016)	DA ns LA ns	DA ns LA ns	DA ns LA ↓	DA ns LA ns	DA ↓ LA ↓	LA OP2 notched	DA ns LA ns	ns	nt
Constable et al. (2020)	LA ns	LA ↓	LA ↓	LA ↓	LA ↓	nt	nt	nr	nt
Constable et al. (2021)	LA ns	LA ns	nt	LA ns	LA ↓	nt	nt	ns	nt
Lee et al. (2022)	LA ns	LA ↓	LA ↓	LA ns	LA ↓	nt	nt	ns	nt
Constable et al. (2022)	LA nr	LA nr	LA nr	a40 ↓	b40 ↓ LA ↓	OP80, OP160 ↓	nt	nr	nt
Friedel et al. (2022a)	LA ns	LA ns	ns	LA ns	LA ns	nt	nt	ns	nt
Attention deficit hyperactivity disorder									
Bubl et al. (2015a)	nt	nt	nt	nt	nt	nt	nt	nt	noise ↑
Lee et al. (2022)	LA ns	LA ↑	LA ↑	LA ns	LA ↑	nt	nt	p72 ↑	nt
Constable et al. (2022)	LA nr	LA nr	LA nr	a20 ↑	b20, b40 ↑	OP80, OP160 ↑	nt	nr	nt
Dubois et al. (2023)	LA ↓♂	ns	LA nr	LA ↓♀	LA ↓♀	nt	nt	ns	nr

Indicative changes indicated by the symbols: ↓ = slower (longer) time to peak or reduced amplitude, ↑ = faster (shorter) time to peak or larger amplitude, DA = Dark adapted, LA = Light Adapted, ns = non-significant. LRF- Luminance Response Function refers to an analysis of either the dark adapted or light adapted (photopic hill) luminance response characteristics of the b-wave amplitude. Findings may not represent all flash strengths and the reader is referred to the study for further details. ns = non-significant, nr = not-reported, nt = not-tested, OPs = Oscillatory Potentials, PhNR = Photopic Negative Response, PERG = Pattern electroretinogram, PT = peak time, RGCs = retinal ganglion cells.

### Autism spectrum disorder

ASD is a neurodevelopmental condition with a reported prevalence of approximately 1–4% with a male bias of approximately 3.8:1 (Baird et al., 2006; Maenner et al., 2023). ASD is characterized by a delay in language, stereotypical repetitive behaviors, and a lack of social reciprocity, whose features have a complex underlying pathophysiology relating to environmental and genetic factors (Piggot et al., 2009; Lai et al., 2014; Lord et al., 2018). The heterogeneric nature of ASD makes the search for a viable biomarker challenging (McPartland, 2021; Parellada et al., 2022) even in large cohorts (Traut et al., 2022).

**Figure 2:**
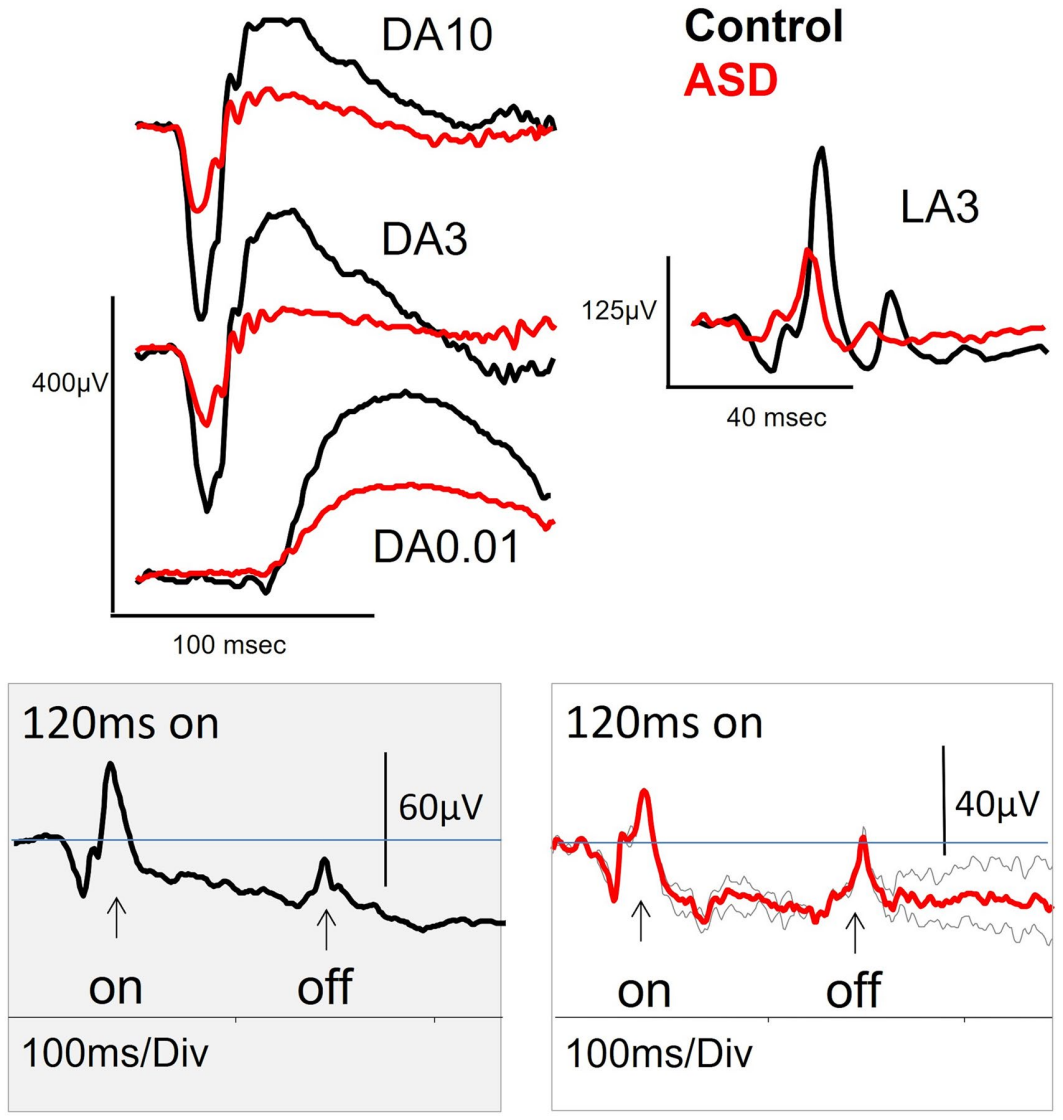
The dark-adapted (DA) and light-adapted (LA) electroretinogram (ERG) waveforms for a control (black) and a high functioning adult individual with ASD (red). The DA3, DA10 and LA3 all exhibit a reduced b-wave amplitude for the ASD subject. Lower panels show the extended LA ON flash of 120 ms that separates the ON-and OFF- pathway components of the ERG waveform (note different amplitude scales). The ASD subject (repeated trials in grey, grand average in red) has a normal OFF pathway response with diminished ON-pathway suggesting the deficit in the b-wave amplitude was the result of a difference in the LA ON-pathway neural generators to the LA b-wave amplitude. Figure modified from Constable et al. (2016).

The first study of the ERG in ASD was conducted by Ritvo et al. (1988) who reported smaller DA b-wave amplitudes in 13 out of 27 ASD individuals with a mean age of 18.9 years compared to 20 age matched controls. The authors also performed a LA flash and DA flicker ERG, but these were not reported, however, Ritvo hypothesized based on the DA findings with a dim red and blue flash that the reduced b-wave may have been due to an anomaly in glutamate signaling but was unable to demonstrate this at the time. Two decades later a smaller study with 11 ASD subjects with a mean age of 37.2 years confirmed a reduced b-wave amplitude under DA conditions (Constable et al., 2016). These authors extended the DA findings by exploring the ERG under LA conditions where a reduced b-wave amplitude was also evident as shown in Figure 2. In addition, the ON-b-wave component of the ON–OFF LA flash ERG waveform was reduced in ASD. The OFF-component was normal supporting a primary ON-pathway deficit in ASD (Constable et al., 2016). The authors also noted some variations in the early LA OPs shape in the ASD group; a bifurcation of OP2 was noted in some individuals suggesting some disturbance in the generation of the OPs. Other findings in this adult population were no significant differences in measures of the DA (15 Hz) and LA (30 Hz) flicker responses nor the PhNR (Constable et al., 2016). The PhNR was also not-significantly different in a larger group of 55 ASD subjects with mean age of 13.6 years suggesting normal RGC function in this young ASD group (Constable et al., 2021) and supported by findings in an adult population (Friedel et al., 2022a). Thus, there is little evidence for RGC functional loss in ASD at this stage.

In a large multicenter study, with 90 ASD subjects with a mean age of 13.0 years, Constable et al. (2020) found a smaller LA b-wave in the ASD group compared to the 87 control subjects, with mean age of 13.8 years. Further mathematical modelling of the LA luminance response function (photopic hill) using the methods of Hamilton et al. (2007) showed a greater loss of the ON-rather than the OFF-pathway in the ASD subjects. However, Friedel et al. (2022a) did not replicate the reduced LA b-wave amplitude in a high functioning adult population using the same stimulus protocol. This may indicate that the observed ERG changes depend in part upon the age and severity of ASD. Also, the heterogeneity in this population and co-occurrence of ADHD for example (Antshel and Russo, 2019) may hinder the identification of a robust biomarker based solely on time-domain features of the ERG waveform. Further studies that may include additional physiological markers, such as the light pupillary response and/or heart rate variability (Daluwatte et al., 2013; Cheng et al., 2020b), which, combined with psychometric profiles may aid in the discovery of an additional early clinical test for ASD. Further studies will also need to explore the case where the diagnosis of ASD occurs with a co-occurring condition to identify the specificity of the biomarker for ASD.

There remain several limitations to the ERG and its application to the early diagnosis and management of ASD that would enable earlier intervention to potentially improve the long-term outcomes of individuals with ASD (Whitehouse et al., 2019; Masi et al., 2021). Most notable would be performing recordings on young children at an age before ASD may be suspected owing to a lack of eye contact or language development by the age of 18 months (Lord et al., 2018) and adequate age and sex matched reference ranges. Furthermore, there is a need for large scale studies that utilize standardized clinical scores based on the Autism Diagnostic Observational Schedule-2 (Gotham et al., 2009; Hus et al., 2014) to fully explore the potential of the ERG as a sensitive measure of clinical severity. As discussed, the prevalence of co-occurring conditions with ASD complicates the ability of the ERG to selectively identify the ASD phenotype when the individual may meet diagnostic criteria for other neurological conditions such as ADHD, but the development of deep learning algorithms and additional methods of signal analysis may enable the ERG to contribute to the earlier diagnosis and improved management of ASD in the future (Mohammad-Manjur et al., 2022; Schulte-Rüther et al., 2023; Tursini et al., 2023).

### Attention deficit hyperactivity disorder

ADHD is more prevalent than ASD but may present as a co-occurring diagnosis with ASD (Bougeard et al., 2021; Kessi et al., 2022). ADHD is characterized by a lack of attention and increased risk taking with difficulty maintaining attention on a given task (American Psychiatric Association, 2013). There is no sex bias, and the prevalence is estimated to be approximately 5% of the population (Polanczyk et al., 2007). ADHD-inattentive subtype or commonly referred to as attention deficit disorder (ADD) do not exhibit the hyperactive behaviors characterized by ADHD. For a recent review on ADHD see Kessi et al. (2022).

**Figure 3:**
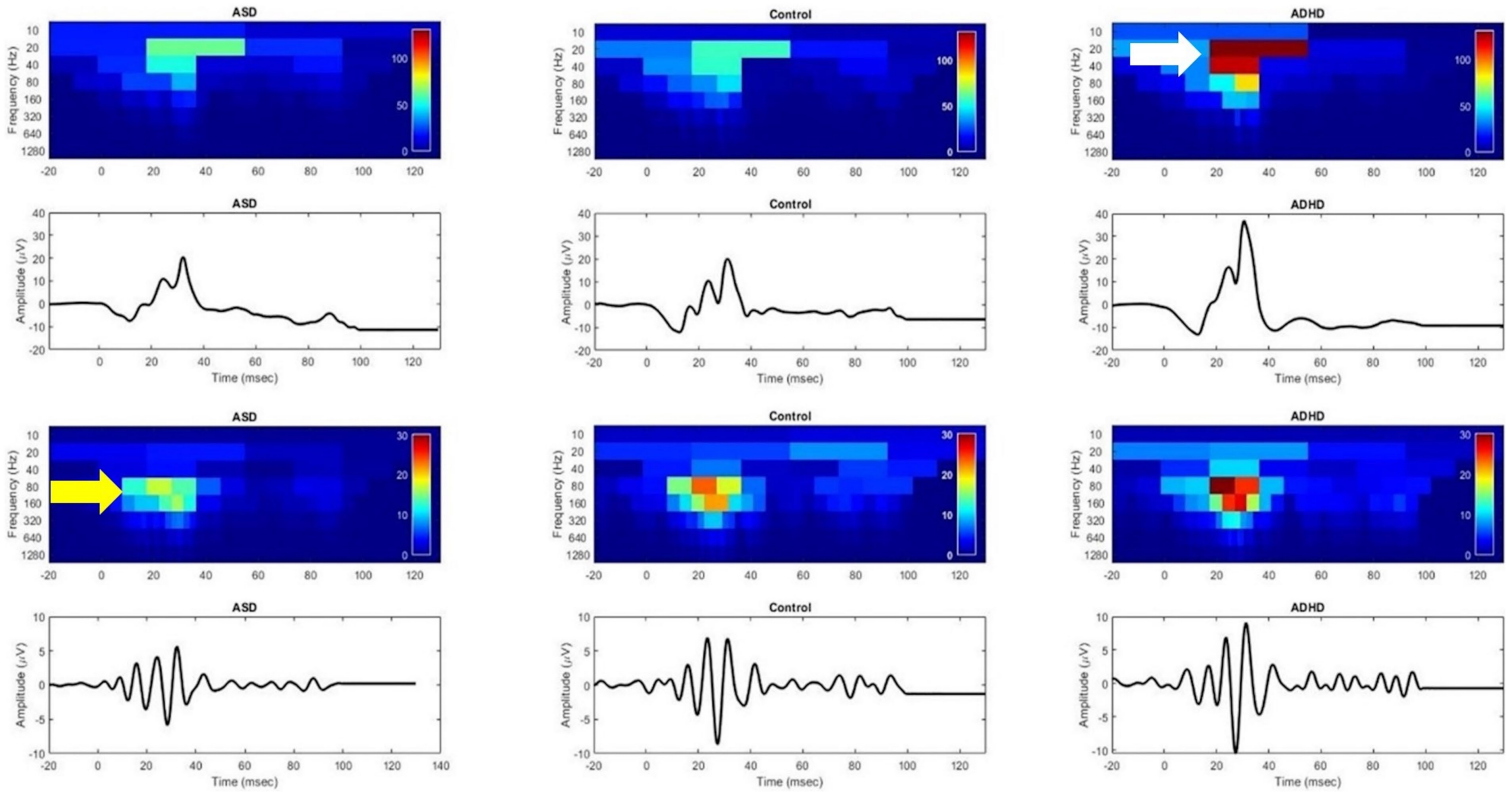
Discrete Wavelet Transform scalograms of energy in the electroretinogram (ERG) waveform for an autism spectrum disorder (ASD), control, and attention deficit hyperactivity disorder (ADHD) child. Colors normalized to ADHD. Upper panel shows the elevated energy in the ERG waveform for ADHD specifically localized to the 20-40 Hz frequency bands which differentiates ADHD from control and ASD (white arrow). Lower panel shows the oscillatory potentials (ERG high-frequency components) with ASD having a lower energy than control and ADHD in the 80-160 Hz bands. This suggests that the loss of high frequency bands is associated with ASD whilst the increased energy in the lower frequency bands is typical of ADHD. Figure modified from Constable et al. (2022).

Visual electrophysiological studies in ADHD have identified an increased background retinal noise that correlates with inattention scores from PERG recordings in 20 non medicated adult ADHD subjects compared to 20 healthy controls (Bubl et al., 2015a). The retinal noise normalized following administration of methylphenidate which increases dopamine in the CNS. This suggests that dopaminergic retinal neurons were responsible for the retinal noise (Werner et al., 2020). To date only small studies have been performed investigating the ERG in ADHD. The first study of the LA ERG in 15 ADHD subjects with a mean age of 15.3 years noted elevated b-wave amplitudes (Lee et al., 2022). Lee et al. (2022) proposed that the larger b-wave amplitude in the ADHD group was due to an imbalance in glutamate and GABA signaling in the retina. Aspects of the OPs were not reported but the modelling of the photopic hill suggested that the ON-pathway may be preferentially disrupted in ADHD. Signal analysis based on the work of Gauvin et al. (2014, 2015, 2017) using discrete wavelet trasform analysis of the LA ERGs in ASD, ADHD and control subjects revealed an interesting “see-saw,” of reduced OP energy in ASD compared to controls and elevated energy in the b-wave and OP components ADHD group compared to controls and ASD. This supports using signal analysis as a method to differentiate these groups due to contrasting differences in the energy of the OPs and b-wave of the LA ERG (Constable et al., 2022; Mohammad-Manjur et al., 2022) see Figure 3. More recently, Dubois et al. (2023), in 26 adult ADHD subjects aged 27.3 ± 4.5 years, found different results between the sexes. The authors reported that the LA a-and b-wave amplitudes were reduced in females with ADHD at flash strengths greater than 7.5 cd.s.m^−2^ and the LA -a-wave time to peak was delayed in male ADHD subjects at the maximal flash strength of 50 cd.s.m^−2^. There was no significant difference under DA conditions for the ADHD group for the pure rod or mixed rod-cone ERGs. Further work is needed in larger study populations with a well-defined clinical severity rating to explore the influence of sex, age, and ocular pigmentation on the ERG findings in ADHD with well-matched controls. This is because it is known that males typically have smaller full field ERGs than women (Brûlé et al., 2007) and ocular pigmentation (Al Abdlseaed et al., 2010) and age (Birch and Anderson, 1992) can affect the amplitude of the ERG.

One further difficulty in exploring the ASD and ADHD populations is that ADHD is often co-diagnosed with ASD (Bougeard et al., 2021; Kessi et al., 2022; Krakowski et al., 2022). In addition, no studies have compared the ADHD with ADD ERG to determine if the hyperactivity component drives the reported ERG changes in the preliminary studies. The main limitation of the studies to date is that they were limited to a specific clinical phenotype of either ASD or ADHD with little known about the effects of co-occurring neurodevelopmental conditions or potential effects of long-term use of psychotropic medications.

### Early adult onset

This section presents the main findings from studies where the central nervous disorders typically present in early adulthood and includes, schizophrenia, bipolar disorder, major depressive disorder, and seasonal affective disorder. Table 3 summarizes the main ERG findings to date with EOG findings summarized in Table 4.

**Table 3:** Summary of findings in studies reported in this review.

	Time to Peak		Amplitude					RGC	
Study	a-wave	b-wave	^‡^Vmax	a-wave	b-wave	OPs	Flicker	PhNR	PERG
Schizophrenia									
Warner et al. (1999)	DA ns LA ns	DA nr LA ns	nt	DA ↓ LA↓	DA ↓ LA ns	DA nr	nt	nt	nt
Balogh et al. (2008)	LA ns	DA↓ LA ns	nt	LA ↓ (acute phase)	LA ns	nt	nt	nt	nt
Hébert et al. (2010) ^†^	LA ns	DA ns LA ns	DA ↓ LA ns	DA ↓ LA ns	DA ns LA ns	nt	nt	nt	nt
Hébert et al. (2015)	nr	LA ↓	DA ↓ LA ↓	DA ↓ LA ↓	DA ↓ LA ↓	nt	nt	nt	nt
Demmin et al. (2018)	DA ↓ LA ns	DA ↓ LA ↓	nt	DA ↓ LA ↓	DA ↓ LA ↓	nt	LA ↓ amp	p72 ↓	nt
Hébert et al. (2020)	DA ns LA ns	LA ↓	LA ↓	DA ↓ LA ↓	DA ↓ LA ↓	nt	nt	nt	nt
Moghimi et al. (2020)	LA ns	LA ns	nt	LA ns	LA ns	LA ns	nt	p72 ↑	ns
Bernardin et al. (2020)	DA ns LA ns	DA ↓ LA ns	nt	DA ↓ LA ↓	DA ↓ LA ns	nt	nt	nt	P50, N95 amp ↓, N95 PT ↓
Bernardin et al. (2021)	nr	nr	nt	nr	nr	OP1-3, DA ↓	nr	nr	nt
Bipolar disorder									
Lam et al. (1997)	DA nr	DA nr	nt	DA nr	DA ns	nt	nt	nt	nt
Balogh et al. (2008)	LA ns	LA nr	nt	LA ns	LA ns	nt	nt	nt	nt
Hébert et al. (2020)	DA ns LA ns	DA ns LA ↓	DA ↓ LA ns	DA ↓ LA ↓	DA ↓ LA ns	nt	nt	nt	nt
Major depressive disorder									
Bubl et al. (2010)	nt	nt	nt	nt	nt	nt	nt	nt	ss contrast gain ↓
Bubl et al. (2012)	nt	nt	nt	nt	nt	nt	nt	nt	ss amp ↓
Fam et al. (2013)	DA ns	DA ns	nt	DA ns	DA ns	nt	nt	nt	ns 0.8°
Hébert et al. (2017)	DA ns LA ns	DA ns LA ↓	LA ns	DA ↓ LA ns	DA ns LA ns	nt	nt	nt	nt
Friedel et al. (2021)	nt	nt	nt	nt	nt	nt	nt	nt	ss 18.75 rps ratio (0.8°/16°) ↓
Cosker et al. (2021)	DA ↓ LA ↓	DA ns LA ↓	nt	DA ns LA ↑	DA ↑ LA ↑	nt	LA PT ↑	nt	P50 PT ↓ N95 ns
Schwitzer et al. (2022a)	nt	nt	nt	nt	nt	nt	nt	nt	P50 PT ↓ N95 PT ↓
Lubiński et al. (2023)	nt	nt	nt	nt	nt	nt	nt	nt	P50, N95 amp ↓
Seasonal affective disorder									
Lam et al. (1992)	DA ns	DA ns	nt	DA ns	♀DA ↓ ♂ DA ↑	nt	nt	nt	nt
Oren et al. (1993)	nt	nt	nt	nt	nt	nt	nt	nt	ns
Hébert et al. (2002)	nr	nr	^#^DA ns	nr	nr	nt	nt	nt	nt
Hébert et al. (2004)	nr	nr	nr	nr	*DA ns	nr	nr	nr	nr
Lavoie et al. (2009)	DA ns LA ns	DA ↓ LA ↓	^#^DA ns LA ↓	DA ns LA ns	DA nr LA nr	nt	nt	nt	nt

Indicative changes indicated by the symbols: ↓ = slower (longer) time to peak or reduced amplitude (amp), ↑ = faster (shorter) time to peak or larger amplitude, DA = Dark adapted, LA = Light Adapted, ns = non-significant. Findings may not represent all flash strengths and the reader is referred to the study for further details. For the dark adapted (DA) condition, V_max_ is defined as the b-wave amplitude at −1.25 log cd.s.m^−2^ (Hébert et al., 2010). ns = non-significant, nr = not-reported, nt = not-tested, OPs = Oscillatory Potentials, p72 = amplitude at time = 72 msec from stimulus, PhNR = Photopic Negative Response, PERG = Pattern electroretinogram, PT = peak time, RGC = retinal ganglion cells, rps = reversals per second, ss = steady state.

† Recorded in children of schizophrenic or bipolar parents.

‡ V_max_ as defined in the studies by Hébert et al. (2017) as being the average of the b-wave amplitudes at flash strengths of 13.3, 23.7 and 50 cd.s.m^−2^ for the LA condition.

# Note that these studies identified a reduced retinal sensitivity based on log K of the Naka-Rushton luminance response function whilst V_max_ was not significantly different.

* Significant group differences to reach 50 μV threshold based on the DA luminance response function.

**Table 4:** Summary of electro-oculogram (EOG) findings in studies reported in this review for bipolar disorder, seasonal affective disorder, and Parkinson’s disease.

Study	LP:DTratio	DT amplitude	LP time
	Bipolar disorder		
Lam et al. (1997)	ns	ns	nr
	Seasonal affective disorder		
Lam et al. (1991)	Winter ↓	nr	nr
Ozaki et al. (1993)	ns	nr	nr
Ozaki et al. (1995)	ns	nr	nr
	Parkinson’s disease		
Economou and Stefanis (1978)	decreased	nr	nr
Ikeda et al. (1994)	decreased (stage 2)	nr	delayed ~4 min
Palmowski-Wolfe et al. (2006)	ns	nr	nr

Indicative changes indicated by the symbols: ↓ = reduced amplitude or slower (longer) time to peak. DT = Dark Trough, LP = Light Peak. LP:DT ratio = Light Peak to Dark Trough ratio, ns = not-significant, nr = not-reported.

### Schizophrenia

Schizophrenia is currently classified as an individual exhibiting at least two or more of the following: delusions, hallucinations, disorganized speech, catatonic behavior, or a diminished emotional expression or avolition (lack of motivation to achieve a goal; Tandon et al., 2013). The median prevalence rate for schizophrenia, which show no sex bias, is estimated to be 4.6/1000 (Saha et al., 2005). Estimates of the co-occurrence of ASD and schizophrenia range from 0 to 36% (Chisholm et al., 2015).

The first report of reduced LA ERGs in schizophrenia was observed in 6 out of 9 patients who had a history of sun gazing. Dopaminergic responsive ganglion and horizontal cells were proposed to be responsible for the reduced ERG amplitudes (Gerbaldo et al., 1992). Further early findings by Warner et al. (1999) indicated significantly reduced a-and b-wave amplitudes under DA conditions in a small sample of 9 subjects but no differences in LA ERGs, nor dark adaptation thresholds and they hypothesized that the preliminary findings may be explained by alterations in fatty acids in the photoreceptors. Individuals with schizophrenia are known to have depleted stores of essential polyunsaturated fatty acids (Zhou et al., 2020) for membrane phospholipids which are found in high concentration in photoreceptors. A larger study by Balogh et al. (2008) compared ERGs from subjects with schizophrenia (*n* = 26), bipolar disorder (*n* = 17) and heathy controls (*n* = 20) with an ISCEV 1989 standard flash strength of 1.7 cd.s.m^−2^ on a 20 cd.m^−2^ background. Between group comparisons were made at baseline (at time of acute presentation where the subjects were hospitalized following psychotic episodes) and at follow-up for the schizophrenia group (at least 2 weeks following medications to enable hospital discharge). The medications for the schizophrenic group included antipsychotics with agonist activity for dopamine and serotonin receptors-including the D2 dopamine receptor: olanzapine [10–25 mg/day, *n* = 12], risperidone [4–8 mg/day, *n* = 6], quetiapine [450–800 mg/day, *n* = 3], and the antiepileptic GABA agonist clozapine [150–400 mg/day, *n* = 5] that structural analysis suggests has affinity for the GABA_B_ receptor (Nair et al., 2020). The bipolar group medications included: olanzapine [10–30 mg, *n* = 9], risperidone [2–8 mg, *n* = 4], valproate [1200–1800 mg/day, *n* = 13], lithium [900–1,200 mg/day, *n* = 4] and clonazepam [0.5–4 mg/day, *n* = 10]. The schizophrenia group, in the acute phase of their condition had smaller LA a-wave amplitudes compared to both the bipolar disorder and control groups. There were no significance differences between the three groups (schizophrenia, bipolar disorder, and control) for the LA b-wave amplitudes or a-wave peak times. Importantly there was recovery of the a-wave amplitude to normal levels following treatment in the schizophrenia group supporting a role for LA a-wave amplitude for differentiating between an uncontrolled and controlled state of schizophrenia. Given no change in the b-wave was observed with medications that target dopamine and serotonin receptors – it was unlikely that the observed ERG changes in the a-wave are a result of dopaminergic effects. The antiepileptic valproate increases regional concentrations of GABA and in the bipolar disorder subjects may be anticipated to increase GABAergic mediated horizontal cell inhibition of the photoreceptors and reduce the a-wave which was not observed in the bipolar group in the acute phase. This may be due to valproate not reaching concentrations in the outer retina to change retinal GABA levels. The authors speculated that the reduction of LA a-wave amplitude in the acute psychotic phase of schizophrenia might be due to the action of fatty acid metabolism as suggested by Warner et al. (1999). The authors highlighted a need for further studies to examine the effects of antipsychotics and antiepileptics on the ERG. The study also highlights the difficulty in interpreting ERG findings when different dosages and pharmacological classes of medications are used in a clinical group. It was also unusual that the LA b-wave amplitude was not commensurately smaller with the a-wave, but the findings provided an important identification of abnormal a-wave morphology in acute schizophrenia.

Hébert’s group have since led many of the studies into the clinical utility of the ERG in discriminating between bipolar disorder and schizophrenia and typically developing individuals (Hébert et al., 2010, 2015, 2020). Extending the luminance response series with stronger and weaker flashes under LA or DA conditions presents a challenge for individuals with this condition. The group therefore first assessed the ERGs in children of families with strong family history of either schizophrenia or bipolar disorder who were designated at high risk of exhibiting traits of schizophrenia or bipolar disorder (Maziade et al., 2005). The pooled results from the siblings of both bipolar and schizophrenia families revealed smaller DA b-wave amplitudes and significantly reduced DA V_max_ (*p* < 0.001). LA-ERGs were within the normal reference range (Hébert et al., 2010). One mechanism proposed was that an increased inhibition through GABA signaling of the horizontal cells could reduce both the a- and b-wave amplitudes through inhibition of the photoreceptors and bipolar cells (Demmin et al., 2018; Hébert et al., 2020). An imbalance between GABA and glutamate neurotransmitters in the prefrontal cortex (Mayeli et al., 2022) and thalamus (Quiñones et al., 2021) has been implicated in schizophrenia which lends support for the suggested GABAergic influence on the ERG by horizontal cells.

In further support of retinal differences in schizophrenia, several studies have reproduced the findings of smaller ERGs in schizophrenic subjects. In the largest study to date of 105 schizophrenic adult subjects and 150 age matched controls Hébert’s group showed both a- and b-wave amplitudes were smaller under both DA and LA conditions, and the LA b-wave time to peak was delayed (Hébert et al., 2015). The strengths of this study were the group sizes and the exclusion of schizophrenics taking medications at the time of testing. In addition, the study used different flash strengths to evaluate the luminance response functions under DA and LA conditions. The main findings were that under LA conditions there was a reduced a- and b-wave amplitude with a delay in the time to peak of the b-wave and reduced peak of the photopic hill. Under DA conditions, there were reduced a- and b-wave amplitude and V_max_ peak of the DA luminance response series. Taken together, these findings support functional retinal responses as a biomarker for schizophrenia. Indeed, it was later shown that the ERG findings could be used to differentiate between bipolar disorder and schizophrenia with an AUC of 0.83–0.86 (depending on medication status) using a larger population of 150 schizophrenics, 151 bipolar disorder and 200 controls that were adjusted for medication use (Hébert et al., 2020). The main difference between schizophrenia and bipolar disorder was the LA b-wave amplitude that was selectively reduced in schizophrenia but not bipolar disorder. Demmin et al. (2018) used skin electrodes to record LA and DA ERGs in 25 schizophrenic and control adult subjects. The group reported a reduced a- and b-wave amplitude and 30 Hz flicker response consistent with previous findings but also identified a reduced PhNR suggesting ganglion cell loss of function in schizophrenia. Further studies using the PERG may help to evaluate the extent of RGC functional loss in schizophrenia in larger clinical populations.

To further extend the potential of identifying individuals at risk of developing schizophrenia, Moreau et al. (2022) used a combination of six ERG factors of amplitude and peak time from DA and LA recordings to predict the risk of psychosis in children whose parents have schizophrenia, opening the possibility of using the ERG to identify individuals most at risk of developing a psychiatric illness. Maziade et al. (2022) assessed 99 subjects aged 5–27 years who had a parent with a diagnosis of either schizophrenia, bipolar disorder, or major depressive disorder. They found a significant (*p* = 0.02) LA b-wave delay of ~1 msec in the undilated cone V_max_ amplitudes for the three included flash strengths (13.3, 23.7 and 50 cd.s.m^−2^) that were used to define the V_max_ of the photopic hill as described by Hébert et al. (2020). The delayed b-wave peak times were associated with lower social functioning and experience of psychotic episodes. These group findings suggest a retinal biomarker may identify individuals at risk of developing psychiatric illness when there is a positive family history of mental illness (Maziade et al., 2022). Bernardin et al. (2021) investigated ERG and PERGs in 29 subjects with schizophrenia and 28 controls and found reduced DA a- and b-waves amplitudes and reported delays in the PERG P50 and N95 peaks. A PERG P50 delay implies some dysfunction of the macular cones. The schizophrenic group were divided into those with a history of visual (*n* = 12) hallucinations or a history of either none or auditory hallucinations (*n* = 17). A greater delay in the DA b-wave peak time was found for the subgroup who had experienced visual hallucinations compared to those that had not, suggesting the DA ERG has potential in identifying subgroups within schizophrenia subjects. These promising findings have the potential to enable early interventions in individuals identified through visual screening to improve their long-term outcomes.

Moghimi et al. (2020) explored possible RGC dysfunction in 30 schizophrenia subjects with a just significantly (*p* = 0.046) larger PhNR recorded at the strongest (7 cd.s.m^−2^) of three tested flash strengths, with greater variability in males. The authors however found non-significant group differences in the PERG, a test of macular RGC function and the LA a- and b-waves in contrast to previous studies (Balogh et al., 2008; Hébert et al., 2010, 2020; Demmin et al., 2018). There were no strong effects of sex on the amplitudes of the PERG or PhNR, but a trend of longer N95 peak time was reported in male schizophrenic subjects. Moghimi et al. (2020) found no significant differences in the LA OPs filtered between 75–100 Hz or 100–300 Hz. Earlier studies had reported mixed results with Raese et al. (1982) reporting a greater variability in OP amplitudes of male subjects, but this was not replicated in a later study by Schechter et al. (1987). More recently, smaller DA OPs (1, 2, and 3) and mfERG amplitudes were reported in 29 schizophrenic patients that suggests possible amacrine cell involvement also in schizophrenia, but the results of the full field DA and LA ERGs for the DA and LA were not reported unfortunately (Bernardin et al., 2021).

Some structural changes at the macula have been reported in schizophrenia, including reduced central macular thickness (Sarkar et al., 2021) and an increased foveal avascular zone (Silverstein et al., 2021). These may explain the localized mfERG functional differences reported in schizophrenia, with normal PERGs that are summed over a larger macular area and are not sensitive enough to be altered by foveal changes (Bernardin et al., 2021). Nonetheless there is promise that structural and functional changes may help to diagnose and monitor individuals with schizophrenia (Silverstein et al., 2020; Prasannakumar, 2023) as well as helping to identify individuals at risk of developing schizophrenia (Demmin et al., 2018; Hosak et al., 2018; Hébert et al., 2020; Komatsu et al., 2022; Maziade et al., 2022; Moreau et al., 2022; Peredo et al., 2022). Taken together, these functional and structural findings suggest that retinal biomarkers will become a useful clinical tool in the identification of individuals at risk of and with schizophrenia to improve management and early diagnosis.

### Bipolar disorder

Bipolar disorder is characterized by episodes of mania followed by either depression or hypomania (American Psychiatric Association, 2013) which have a significant negative impact on the individual and their families (Khafif et al., 2021). The prevalence of bipolar disorder is estimated at approximately 1% of the population (Vieta et al., 2018).

The first exploration of the ERG and EOG findings in bipolar disorder were performed by Lam et al. (1997) who found no differences in the EOG or DA-ERG parameters suggesting no dysfunction in the RPE-rod-bipolar cell pathway in 24 bipolar disorder subjects on long-term lithium use. Subsequently, Balogh et al. (2008) reported no significant differences in LA ERG in 17 individuals with bipolar disorder. The largest study to date involving 151 bipolar subjects by Hébert et al. (2020) confirmed a normal LA b-wave amplitude and showed that the LA b-wave amplitude could distinguish individuals with bipolar disorder from those with schizophrenia with 80% sensitivity and 82% specificity. The authors also reported smaller DA a- and b-wave amplitudes in bipolar disorder.

Children at risk of developing either bipolar disorder, major depression, or schizophrenia owing to a parent being diagnosed with one of these conditions are reported to show slower LA b-wave peak time and a smaller DA b-wave amplitude which suggests the ERG may help predict the transition to adult disease (Maziade et al., 2022). Additional findings in children of a parent with schizophrenia or bipolar disorder identified two clusters, those with ERGs within normal reference range and those with reduced DA b-wave amplitudes and delayed LA b-wave time to peak. The group with abnormal ERGs were at a higher risk of cognitive dysfunction in domains of processing speed, verbal memory, visual and working memory and executive function (Peredo et al., 2020). These findings of slower LA b-wave, smaller DA a and b-waves suggest that bipolar disorder may be characterized by disruption to the phototransduction cascades in rods and cones or inhibitory horizontal cells (Barnes et al., 2020; Hirano et al., 2020) or feedback of the OFF-pathway inputs that reduce the a-wave amplitude (Bush and Sieving, 1994; Dang et al., 2011). Future large scale clinical studies are planned to further identify retinal biomarkers in conjunction with sleep parameters in bipolar disorder that may help with the earlier diagnosis of this condition (Gross et al., 2022).

### Major depressive disorder

Depression and anxiety can form a part of any mental health condition but can also be clinically significant in isolation with a worldwide prevalence estimated at 2.6% (Polanczyk et al., 2015). GABA regulation has been implicated in its pathophysiology (Kalueff and Nutt, 2007; Athira et al., 2020). Major depressive disorder is characterized by poor mood and reduced motivation to find pleasurable experiences and may also be accompanied by physical symptoms such as insomnia and poor concentration (American Psychiatric Association, 2013).

Fam et al. (2013) reported no significant differences in the DA a- and b-wave amplitude or peak times in 20 subjects with depression compared to 20 age matched controls. They did note a loss of Landolt C contrast sensitivity, but concomitant PERG recordings to 0.8° check widths of a range of contrasts 7–63%, suggested that the retinal macular contrast function was normal. The authors commented that the visually evoked potential might have identified if the reduced contrast sensitivity was the result of higher cortical functional loss. A subsequent larger study by Hébert et al. (2017) compared ERG parameters of 100 subjects with major depressive disorder, of which 17 were non medicated, to 100 controls. They found smaller LA a- and b-wave amplitudes in the non-medicated subgroup, which suggests that the antidepressant medications taken may help to normalize the LA ERG. The medicated group included mainly individuals taking a Selective Serotonin Reuptake Inhibitor (*n* = 35); Serotonin-Norepinephrine Reuptake Inhibitor (*n* = 32); antipsychotics (*n* = 36) or a mood stabilizer (*n* = 19). Antipsychotics mostly block dopamine receptors, and the normalization of the ERG supports a role for the ERG in determining the efficacy of dopaminergic medications in this group where some individuals may respond more than others to treatment (Fornaro et al., 2011). Few studies have evaluated the effect of serotonin on the ERG, with mixed effects reported in animal studies. Cunningham and Neal (1983) reported no effects of serotonin on the DA ERG in rabbit. Whilst in frog, the DA b-wave amplitude is increased by serotonin (Popova and Kupenova, 2017). In a mouse two separate models Lavoie et al. (2014b) showed an increase in the b-wave implicit time in a R439H tryptophan hydroxylase 2 knockin (Tph2-KI) mouse that decreased cortical serotonin by 80% but had no effect on retinal serotonin levels. However, the dopamine transporter knock out mouse (DAT-KO) had a reduced DA retinal sensitivity and supports a more likely effect of dopamine on the ERGs in depression. Therefore, it is uncertain how serotonin reuptake inhibitors may influence the ERG in human, given the different effects on the ERG in rabbit, mouse and frog studies to date.

Moulard et al. (2022) assessed retinal function using the ERG, PERG and mfERG in major depressive patients at baseline and 4-, 8-and 12-weeks following treatment with either a Selective Serotonin Reuptake Inhibitor, Serotonin-Norepinephrine Reuptake Inhibitor, tri-cyclic antidepressant, or a benzodiazepine. The main aim of this preliminary study was to determine if different classes of anti-depressive psychotropic medication would affect the ERG findings. For the group taking a Selective Serotonin Reuptake Inhibitor (*n* = 37) increasing serotonin activity compared to those not (*n* = 19) the PERG P50 was significantly larger and later, whilst the LA3 b-wave peak time was faster. The opposite effect was found for the group taking a Serotonin-Norepinephrine Reuptake Inhibitor (*n* = 15) which prolongs the effect of serotonin and norepinephrine at the synapse increasing post synaptic receptor activation and neuronal activity compared to those not taking this medication (*n* = 41). Here the PERG P50 was smaller and faster and the LA3 b-wave peak time delayed compared to the non-medicated group. These different effects suggest that the PERG or b-wave time to peak may be a potential biomarker with which to monitor the effects of these classes of anti-depressives. For the group taking tricyclic antidepressants (*n* = 9) compared to those not (*n* = 47) the DA3 a-wave peak was delayed, whilst PERG P50 was smaller and faster which is consistent with the findings of the group taking the Serotonin-Norepinephrine Reuptake Inhibitors. Thus, the DA3 a-wave may be more effective at monitoring tricyclic antidepressant efficacy. For the benzodiazepine group (*n* = 10) and those not taking a benzodiazepine (*n* = 22), the 30 Hz flicker amplitude was elevated suggesting this measure may be specific for benzodiazepines. In contrast the Selective Serotonin Reuptake Inhibitor group had smaller 30 Hz amplitudes compared to the comparison group. Taken together the results indicate different effects of antidepressant medications on the PERG, DA3, LA3 and 30 Hz flicker amplitude that may help in the monitoring and treatment of patients with depression depending on the class of medication.

A study by Schwitzer et al. (2022a) compared transient PERG from 24 major depressive subjects to 29 controls interestingly using signal analysis to extract features whose distributions best categorized the groups using a machine learning model. Delays in the P50 and N95 peak times were able to discriminate between groups at baseline and became normalized following luminotherapy over a 12-week period providing the possibility of using PERGs to monitor therapeutic interventions over time (Schwitzer et al., 2022a). The authors highlight the need for larger samples and further investigation of the specificity of the PERG in identifying depressive patients from other psychiatric disorders such as bipolar depression.

Cosker et al. (2021) examined retinal function using full field ERGs, PERGs and mfERGs in a group of 14 adults in the acute phase of major depressive disorder compared to 14 age and sex matched controls. They found the mixed rod cone DA3 flash a-wave peak time delayed and b-wave amplitude elevated, but no group differences in the DA0.01 rod driven b-wave amplitude or time to peak. This suggested that the difference was associated with cone involvement in the mixed DA3 ERG. Indeed, under LA cone mediated conditions the LA3 a- and b-waves peak times were delayed and had a higher amplitude than the comparison group. In addition, the LA3 30 Hz flicker peak times were also delayed but there were no significant differences in the amplitude suggesting abnormal cone kinetics during phototransduction or possibly modulation by inner retinal neurons that are believed to contribute to similar electrophysiological findings in early diabetic retinopathy (McAnany and Park, 2019). The PERG P50 peak time was delayed, and the mfERG second ring amplitude was reduced. Cosker et al. (2021) proposed that the retina was hypersensitive to light based on the findings of elevated LA3 b-wave and further suggested the decreased second ring mfERG amplitude and delay in the P50 peak time reflect a delay in the signaling within the central retina.

Fam et al. (2013) observed reduced Landolt contrast sensitivity but normal transient PERG contrast sensitivity in major depressive disorder. Whilst using ssPERG, Bubl et al. (2010) found reduced contrast sensitivity gain in 20 subjects either medicated or not, with major depressive disorder of 1.1 μV/% change in contrast compared to 3.9 μV/% change in contrast for controls with checkerboard contrast ranging from 3.2–80%. Bubl et al. (2015b) then compared these ssPERGs with pattern visual evoked potentials (pVEPs) and found a significant reduction in the pVEP amplitudes with a greater loss in the contrast transfer function of the PERG (~50%) compared with the VEP (~25%). Although measures of both retinal and cortical function were reduced, the authors conclude that these effects were more apparent in the retina and may be attributed to an imbalance in the dopaminergic system. Some prior support that dopamine is responsible for the reduced ssPERG amplitudes was shown by Bubl et al. (2012) who normalized the ssPERG amplitudes across contrast levels following treatment with various antidepressant medications in 14 subjects. The different combinations of psychotropic medications taken made it difficult to ascribe a particular neurotransmitter to the restoration of the PERG amplitude but included benzodiazepines, Selective Serotonin Reuptake Inhibitors, tricyclic antidepressants, and lithium.

In one recent pilot study in a small sample of 12 subjects with major depression the ratio of the ssPERG amplitudes to different check sizes (0.8°, 1.6°, 3.2°, and 16°) presented at either 18.75 reversals per second (rps) or 12.5 rps were compared with 12 age and sex matched controls (Friedel et al., 2021). The study found significantly reduced ssPERG amplitudes to the smallest standard check size of 0.8° at both reversal rates in the depression group and that the PERG ratio of the 16°/0.8° check size at the higher 18.75 rps rate were significantly different between groups. The authors propose further studies are required and suggest the reduced PERG amplitudes at the smaller check sizes may be due to retinal dopamine levels affecting receptive field size.

Most recently, Lubiński et al. (2023) examined structure–function correlations using transient PERG and OCT in 25 untreated major depressive adults compared to 25 typical individuals. Both the PERG P50 and N95 amplitudes were reduced. The N95 amplitude, which originates in the ganglion cells, was the more frequently abnormal in 32% of eyes and this correlated with the Hamilton Scale scores for depression. It is not clear if this was a selective N95 reduction as the N95:P50 amplitude ratios were not presented. The OCT structural measures of the retina were normal suggesting functional changes may precede any measurable structural changes in depression.

The promising early findings with antidepressant medications on specific aspects of ERG recordings such as the PERG (Moulard et al., 2022) or non-therapeutic interventions using light therapy (Schwitzer et al., 2022a) provide new methods with which to monitor the efficacy of treatments for major depressive disorder. The lack of a structural abnormalities observed in OCT may be due to the condition manifesting in adulthood when the retina is mature, and the subtle or early functional changes are not associated with a manifest change of macular structure.

### Seasonal affective disorder

Seasonal affective disorder is associated with depression during reduced daylight in the winter months and may affect up to 1–2.5% of adults (Blazer et al., 1998; Magnusson and Boivin, 2003). Seasonal affective disorder’s pathophysiology has been linked to serotonin levels that fluctuate with the seasons (Tyrer et al., 2016a,b).

Several studies have examined physiological responses of the eye in winter and summer time in individuals with seasonal affective disorder. Lam et al. (1992) were the first to report a more pronounced reduction of the DA b-wave amplitude in female subjects in a study group of 6 males and 18 females. However, Oren et al. (1993) found no differences in color vision, dark adaptation, pupil diameter or PERG amplitudes in a large sample of 61 subjects measured in the winter and summer months and suggested there was an absence of marked functional retinal changes in seasonal affective disorder. In contrast, Hébert et al. (2002) analyzed the DA luminance response functions recorded in summer and winter to obtain V_max_ (saturated b-wave amplitude) and log K (a measure of retinal sensitivity), in 12 subjects who were compared to 9 controls. In winter the seasonal affective disorder subjects had lower log K than controls but there were no group differences in V_max_. These findings albeit from a small group suggest subjects with seasonal affective disorder subjects have lower retinal sensitivity to light during the winter months. A follow up study estimated the reduction in retinal sensitivity in 55% of the 27 seasonal affective disorder subjects was 1 standard deviation below 23 healthy subjects (Hébert et al., 2004). Hébert hypothesized that this seasonal difference may be due to alterations in the neuromodulators, melatonin and serotonin that can modulate mood (Ahmad et al., 2023).

To determine if retinal sensitivity could be changed with therapy Lavoie et al. (2009) studied the DA and LA b-wave luminance response functions in 22 seasonal affective disorder subjects during the winter/autumn and summer months following 2–4 weeks of light-therapy. The authors found the LA V_max_ and DA log K (retinal sensitivity) of the seasonal affective disorder subjects was reduced in winter, (at baseline) compared to the controls but there were no significant differences between groups in summertime following light therapy. They also measured salivary serotonin concentrations to test the melatonin hypothesis for changes in retinal sensitivity but found no significant differences. This suggests these differences may be due instead to dopaminergic pathways rather than melatonin and serotonin.

Changes in the EOG in seasonal affective disorder have been observed in winter. Lam et al. (1991) was the first to report a reduced light peak to dark trough ratio (LP:DTratio) in 19 unmedicated subjects with seasonal affective disorder. Ozaki et al. (1993) found a trend (when an outlier was removed) for a slightly reduced LP:DTratio in 16 seasonal affective disorder subjects in winter, that were unchanged by 1 week of light therapy. Ozaki et al. (1995) then repeated the study on 16 seasonal affective disorder subjects and 16 controls recording the EOG in winter and then in summer. Surprisingly they found the control group had a significantly higher LP:DTratio in winter compared to summer but there were no significant differences in the seasonal affective disorder group. The authors suggest healthy subjects can adjust and adapt naturally to light whilst subjects seasonal affective disorder cannot and show a constant LP:DTratio throughout winter and summer. Ozaki and colleagues proposed that serotonin levels may be responsible for the lower LP:DTratio, given fluctuations in the LP:DTratio have been observed in normal subjects due to circadian rhythm with larger LP:DTratios at midnight compared to midday (Anderson and Purple, 1980). Table 4 summarizes the findings of the EOG in adult and late adult-onset conditions.

The electrophysiological results with seasonal affective disorder are mixed but provide some evidence for a reduced EOG light rise in the winter (Lam et al., 1991; Ozaki et al., 1993). The ERG b-wave luminance response function parameters differences may help evaluate light-based therapies (Lavoie et al., 2009).

### Late adult onset

Parkinson’s disease (PD) and Alzheimer’s disease (AD) are neurodegenerative disorders that may be characterized by changes in the electrophysiological signals in the visual pathways as part of processes associated with dementia (Douglas et al., 2021). Several structure–function studies have been performed and the results appear dependent on the stage of the disease and interactions with systemic dopaminergic medications (Masson et al., 1993; Garcia-Martin et al., 2014; Kaur et al., 2015; Nowacka et al., 2015).

PD severity is usually defined using the modified severity scale of Hoehn and Yahr (1967), this ranges from early stage 1 to severe stage 5 when individuals are confined to bed. Early stage 1 present with unilateral involvement with limited loss of function, in stage 2 (mild) subjects exhibit bilateral or midline impairment without loss of balance and stage 3 (moderate) is characterized by impaired balance. By the most severe stage 5, subjects are confined to bed. For AD, the severity is based on the Functional Assessment Staging of Alzheimer’s Disease which ranges with increasing severity from 1 to 7 depending on the degree of loss of control of voluntary and involuntary motor functions and memory (Reisberg, 1988). Table 5 summarizes the ERG results in human studies for AD and PD.

**Table 5:** Summary of findings in studies reported in this review.

	Time to Peak		Amplitude			
Study	a-wave	b-wave	a-wave	b-wave	mfERG/OPs	PERG/PhNR
Alzheimer’s disease						
Katz et al. (1989)	DA ns	DA ns	nr	DA ns	nt/nt	P50 amp ↓/nt
Trick et al. (1989)	nt	nt	nt	nt	nt/nt	P50 amp ↓ 4rps and > 16rps/nt
Justino et al. (2001)	DA ns LA ns	DA ns LA ns	DA ns LA ns	DA ns LA ns	nt/DA ns	nt/nr
Parisi et al. (2001)	nt	nt	nt	nt	nt/nt	P50-N95 amp ↓ P50-N35 amp ↓ N35, P50, N95 PT ↓/nt
Kergoat et al. (2002)	nt	nt	nt	nt	nt/nt	ns/nt
Krasodomska et al. (2010)	nt	nt	nt	nt	nt/nt	P50, N95 amp↓ P50 PT ↓/nt
Sartucci et al. (2010)	nt	nt	nt	nt	nt/nt	amp and PT ↓ (Y-Bk)/nt
Moschos et al. (2012)	nt	nt	nt	nt	central ring amp ↓	nt
Ngoo et al. (2019)	nt	nt	nt	nt	nt/nt	P50, N95 amp ↓ P50 PT ↓/nt
Mavilio et al. (2020)	nt	nt	nt	nt	nt/nt	ssPERG amp and phase ↓/nt
Sen et al. (2020)	nt	nt	nt	nt	central ring amp ↓	nt/nt
Asanad et al. (2021)	DA ns LA3 ↓	DA ns LA3 ↓	DA ns LA ns	DA ns LA ns	nt/nt	nt/p72 ↓
Parkinson’s disease						
Nightingale et al. (1986)	LA ns	LA ns	LA ns	LA ↓	nt/nt	P50 amp↓ ♂ > ♀. P50, N95 PT ns/nt
Gottlob et al. (1987)	DA ns LA ns	DA ns LA ns	DA ns LA ↓	DA ↓ LA ↓	nt/nt	P50 amp ↓ 50% contrast/nt
Calzetti et al. (1990)	nt	nt	nt	nt	nt/nt	P50 amp ns P50 PT↓ at high spatial frequency/nt
Peppe et al. (1995)	nt	nt	nt	nt	nt/nt	P50 PT↓ 47 and 96% contrast/nt
Tagliati et al. (1996)	nt	nt	nt	nt	nt/nt	ssPERG attenuated 50% contrast/nt
Sartucci et al. (2003)	nt	nt	nt	nt	nt/nt	B-Y, R-G Y-Bk P50 P50 amp ↓, Y-Bk P50 PT ↓/nt
Palmowski-Wolfe et al. (2006)	ns	ns	ns	ns	ns: early PD (*n* = 4)/nt	ns
Garcia-Martin et al. (2014)	nt	nt	nt	nt	nt/nt	P50 amp↓ P50 PT ↓ N95 amp↓ N95 PT ns/nt
Kaur et al. (2015)	nt	nt	nt	nt	nt/amp and time ↓	nt/nt
Nowacka et al. (2015)	DA ns LA ns	DA ns LA ns	DA ↓ LA ns	DA ns LA ↓	nt/DA (OP1 + OP2 + OP3) amp ↓	nt/nt
Mello et al. (2022)	DA nt LA ns	DA nt LA ns	DA nt LA ns	DA nt LA ↓	nt/LA OPs↓ amp and time	nt/amp ↓

Indicative changes indicated by the symbols: ↓ = slower (longer) time to peak or reduced amplitude (amp). ♂ = male, †Flicker ERGs (4–30 Hz) with B-Y Blue-Yellow gratings, Bk-Y = Black-Yellow gratings, ns = not-significant, nr = not-reported, OPs = Oscillatory Potentials, p72 = amplitude at time = 72 msec, PT = peak time, PERG = Pattern Electroretinogram, PhNR = Photopic Negative Response, rps = reversals per second, ss = steady state.

### Alzheimer’s disease

Although a loss of RGC function in AD was suggested by Katz et al. (1989) after they found a smaller P50 compared to controls in a small sample of 6 individuals with AD. Care is needed to interpreting PERGs in this older age group who may have co-occuring age related maculopathy that has increasing reported prevalance ~20–40% between ages 70-85 years. The PERG P50 is driven by macular cone function and the PERG N95 is selective reduced in RGC dysfunction. It is important to note that attributing specific RGC dysfunction requires a relative greater loss of the N95 components. A N95:P50 amplitude ratio of 1 or less shows selective RGC loss and the P50 peak time can be shorter if RGC loss is severe (Holder, 2001; Luo and Frishman, 2011; Bach et al., 2013). Katz et al., 1989 did not find any significant differences in the DA full field ERG or pVEP parameters compared to controls. Trick et al. (1989) recorded the PERG and pVEP at low transient 4 rps and high steady state 16 rps in 13 elderly subjects with senile dementia of the AD type and confirmed a reduced P50 amplitude which was more pronounced at the faster reversal rate and considered this to support a loss of RGC function in this group. The pVEPs were more affected at the higher reversal rate too and showed no difference at the lower reversal rate. No timing differences were observed in the VEPs, and the authors suggested the deficit was not due to conduction delay, but a degeneration or loss of RGC axons associated with information processing at high temporal rates.

Other studies have probed early RGC loss of structure and function in AD using OCT imaging of the macula and PERGs. Parisi et al. (2001) were the first to explore correlation with PERG and OCT imaging of the macula in 17 subjects with mild impairment of cognitive ability that met diagnostic criteria for AD confirmed by MRI imaging (mild to moderate levels of cortical and cerebral atrophy) and clinical diagnostic instruments. The group found smaller PERG P50 and N95 amplitudes and delayed peak times, but reported that the N95:P50 amplitude ratio correlated with thinning of the retinal nerve fiber layer, supporting RGC axonal loss in AD. It should be noted that at the time of the study accurate segmentation of the retinal layers was limited with the available OCT instruments and software but the thinning if the nerve fiber layer was interpreted as a corresponding loss of RGCs given the reduced PERGs recorded in the AD subjects.

In contrast, Kergoat et al. (2002) studied PERG and pVEPs presented at 1 and 8 rps in 27 mild to moderate dementia of AD type subjects compared to 27 age matched controls and found no differences in the PERG amplitude or peak times but found a delay in the pVEP P100 peak time. The authors suggested that the difference in the results may be due to the clinical severity of this population being mild to moderate and may not have been severe enough to demonstrate a reduction in retinal function. In a follow up study using the same clinical population the group explored potential changes in inner retinal function using the full field DA and LA ERGs but found no significant differences in the waveforms supporting normal inner retinal function at these early stages of AD (Justino et al., 2001).

Krasodomska et al. (2010) also recorded PERGs at 4.7 rps and pVEPs at 2 rps in 30 subjects with early stage AD recently diagnosed in the previous 6 to 24 months. In common with others this group reported, smaller P50 and N95 amplitudes and a delay in the P50 peak time. A delayed P100 peak time in the pVEP was also found with no significant differences in the amplitudes of the VEP parameters. Taken together the results supported a loss of macular cone function that drives the PERG with corresponding slower conduction time along the retino-cortical pathways in this early-stage AD population. Ngoo et al. (2019) also performed PERGs and pVEPs with more refined OCT image analysis in 25 unmedicated early-stage AD subjects. Their findings were consistent with those of Krasodomska et al. (2010) examined the differences in conduction tme along the retinocortical pathway by comparing the differences in the time to peak of the pVEP P100 and PERG P50 in 30 early AD subjects (diagnosed between 6 and 24 months prior to the study) and 30 age matched controls. The findings showed reduced P50 and N95 amplitudes and a delay in the P50 peak time with a similar delay in the pVEP P100 peak time but no significant differences in the amplitude of the VEP between the early stage AD subjects and controls. There was a significantly longer retinocortical conduction time in the AD subjects by approximately 7 msec. Using a different protocol, Mavilio et al. (2020) used a grating pattern to record “re-test” ssPERGs in age matched subjects with vascular dementia (*n* = 16), early-stage AD (*n* = 17) and controls (*n* = 19). The re-test ssPERG, uses five consecutive ssPERG stimulations which are then evaluated based on the within test phase variability of the second harmonic (Mavilio et al., 2015). Between the three groups, the authors found a greater variability in the phase variability in the AD group when comparing the 5 replicates and a significant reduction in the re-test ssPERG amplitudes between AD and controls. They found no significant differences between the vascular dementia group and controls suggesting the retinal functional changes are related specifically to AD.

Sartucci et al. (2010) in newly diagnosed individuals with AD (<1 month), and before any treatment investigated the parvocellular and magnocellular pathways using equiluminant colored sinusoidal gratings of red-green, or blue-yellow (chromatic) and yellow-black (luminance) stimuli, respectively. They recorded transient PERGs and pVEPs in 15 AD subjects and 10 age matched controls naming the waveform P1 (equivalent to P50) and N1 (equivalent to N95). The group reported evidence for a more pronounced magnocellular deficit based on reduced and delayed PERG P1 and N1 peaks in the luminance channel (yellow-black). There were also significant reductions in the P1 amplitude (red-green) and N1 (blue-yellow) chromatic stimuli but no significant group effects on the peak time of these chromatic stimuli. The VEPs also highlighted a likely magnocellular deficit with reduced and delayed luminance response for the N-wave (P100 equivalent) but no differences in the chromatic VEP peak times or amplitudes. The authors suggest that the larger cells involved with the magnocellular pathway may be preferentially affected in AD which may explain the greater reduction of PERGs and pVEPs at mid contrast levels. OCT imaging supports a magnocellular loss in AD with preferential RNFL thinning in the superior and inferior quadrants (La Morgia et al., 2017) but further functional studies of the magnocellular pathway such as loss of contrast sensitivity at low spatial frequencies may help to identify early changes in AD (Skottun and Skoyles, 2007).

Further support for loss of central visual function in AD has been shown in mfERG studies of cone luminance responses. These report decreases of central ring amplitudes that correlate with thinning at the macula caused by retinal nerve fiber loss. Moschos et al. (2012) recorded mfERGs and retinal nerve fiber layer thickness using OCT in 30 AD subjects and found significant thinning of the superior and inferior retinal nerve fiber layers and reduced mfERG p1 amplitudes in the fovea and parafoveal area providing structural evidence of ganglion cell axonal loss associated with functional loss of cone driven macular function. Sen et al. (2020) also recorded mfERG, pattern reversal VEPs and central OCT imaging in 20 AD subjects within 2 years of diagnosis (mild to moderate severity) and found reduced central mfERG amplitudes replicating the findings of Moschos et al. (2012) with corresponding thinning of the superior and inferior retinal nerve fibre layers. Sen et al. (2020) also reported a reduced and delayed P100 pVEP in their population reflecting dysfunction of the retino-cortical pathway. To evaluate retinal electrophysiology as a potential biomarker for pre-clinical AD, Asanad et al. (2021) recruited 14 individuals with no cognitive dysfunction but elevated amyloid ß and 15 age matched controls and recorded PERGs and ERGs to evaluate the potential for the ERG to classify pre-clinical AD. Unlike previous studies, there were no significant differences in the PERG, but the pre-clinical AD group did have smaller PhNR p72 amplitudes-a measure of global RGC function. There were also delays in the LA3 a- and b-wave time to peak, but the p72 amplitude was the best marker for early AD (Asanad et al., 2021).

Of the findings to date, the PERG and mfERG offer the most likely functional tests of central vision that together with OCT imaging of retinal tissue and vascular networks may provide the potential for the earlier diagnosis and management of AD through its disease progression with accumulation of amyloid plaques (Mirzaei et al., 2020). Further testing of changes in magnocellular pathways (Sartucci et al., 2010) using mid yellow-black stimuli or re-test PERGs (Mavilio et al., 2020) or PhNR (Asanad et al., 2021) may lead to tests that can identify AD early and assist with management of this condition.

### Parkinson’s disease

PD results from damage to the substantia nigra and leads to a progressive loss of dopamine in the brain, causing characteristic motor symptoms such as tremor, rigidity, and bradykinesia (Kalia and Lang, 2015). The lifetime risk of being diagnosed with PD is increasing and is more likely in males with an estimated prevalence rate of 0.8% by 2030 in France (Wanneveich et al., 2018) and an age and sex adjusted overall incidence 13.4 per 100,000 (Van Den Eeden et al., 2003). As with AD, the degree of visual functional and structural changes with PD are associated with disease duration, severity, and the interactions of medications. A pre-ISCEV standard study by Nightingale et al. (1986) compared full field ERG, PERG, and pVEP from 36 PD subjects including moderately (*n* = 16) and severely (*n* = 13) affected PD individuals compared to 28 age matched controls. They found smaller pVEP P100 amplitudes particularly in male subjects, but no group differences in the P100 peak time. The PERG P50 amplitude was reduced at a group level with males having a more significant reduction than females. There were no group differences the PERG peak times. The LA b-wave amplitude was smaller at the stronger of two flash energies (tested at 0.1 and 1.0 Joules), but all other LA ERG amplitudes and peak times were comparable with controls. The authors did not perform a DA ERG series, but the results of the PERG and pVEP indicated a loss of central macular cone driven retinal function. Gottlob et al. (1987) also reported reduced a-wave amplitudes under LA conditions and reduced b-wave amplitude under LA and DA conditions but no significant effects in the peak time of the a- and b-waves in 25 PD subjects. They also confirmed a reduced PERG amplitude using a mid-contrast (50%) for the chequerboard stimulus in the PD subjects.

Calzetti et al. (1990) simultaneously recorded PERG and pVEPs to three, high contrast chequerboard spatial frequencies (0.87, 1.74, and 2.44 cycles/degree) presented at transient (5.4 rps) and steady state (8 and 16 rps). The data from 9 subjects with early stage 2 and 3 PD compared to 12 age matched controls showed a significant delay in the PERG P50 peak time only to the transient high spatial frequency pattern, all other PERGs were normal. The pVEP P100 amplitude was reduced, and peak time delayed at the higher spatial frequencies for both transient and steady state stimuli. The findings suggest the early changes in the visual pathways in PD may affect RGCs unusually tuned to higher spatial frequencies and fast temporal processing. Peppe et al. (1995) reported a statistically significant recovery of delayed or non-detectable transient PERGs in 13 newly diagnosed (13 ± 5.6 months) PD individuals after levodopa treatment at medium 47% contrast (but not high 96% contrast) with square wave gratings (2.0 cycles/degree) presented at 2 rps. Peppe et al. (1998) went on to evaluate the effects of spatial frequency in PD on the ssPERG to a sinusoidal grating modulated at 8.55 Hz at 50% contrast with 4 spatial frequencies: 0.6, 2.0, 2.7 and 4.0 cycles/degree. There were three study groups with an age disparity. These were a group of mild to moderate PD (*n* = 18, mean age 60.1 years), a group with post traumatic cortical lesions and clinical symptoms like PD (*n* = 14, mean age 14.1 years) and healthy controls (*n* = 12, mean age 26.8 years). The study identified two key findings. One was that the PERG amplitude in PD was reduced in the mid spatial frequency range of 2.7–4.0 cycles/degree, this was not present in the post trauma group. Secondly the recovery of PERGs with levodopa supports a dopaminergic origin for the PERG amplitude loss in PD. In further support of these findings, Tagliati et al. (1996) recorded ssPERGs to a range of spatial frequencies from 0.5 to 6.9 cycles/degree at 50% contrast with counterphased sinusoidal gratings modulated at 7.5 Hz in 20 PD patients and 20 age matched healthy controls and also identified a loss of ssPERG amplitude to the mid spatial frequencies that was less marked in subjects using levadopa.

Given the more pronounced effects in PD for PERG to mid contrast stimuli, Sartucci et al. (2003) explored the RGC populations related specific visual pathways for the parvocellular RGCs (red-green), koniocellular (blue-yellow) and achromatic (yellow-black) magnocellular pathways with sinusoidal gratings in 12 early PD patients who had not yet started levodopa treatment. The authors found that the P50 amplitudes were smaller (by 50%) for all chromatic and luminance stimuli but there was also a significant P50 peak time delay of 15 msec evident only for the blue-yellow grating. This suggested a more profound loss of function in the koniocellular pathway in early stages of the disorder. Follow up VEP studies using the same stimuli also found a similar pattern of results with a greater delay of P100 peak time in PD for blue-yellow stimuli (Sartucci et al., 2006; Sartucci and Porciatti, 2006).

In one of the largest studies to date Garcia-Martin et al. (2014) investigated structure function corelates between the transient PERG, pVEP, and OCT of the macula and retinal nerve fibre layer in 46 PD individuals in the treated early to moderate stage and 33 age matched controls. Confirming previous findings these authors found involvement of macular cone driven pathways and RGCs, evidence by a reduced and delayed P50 amplitude and peak time and reduced N95 amplitude in the PD group with high contrast (90%) chequerboard stimul (30 min of arc) presented at 2 Hz. However, the N95:P50 amplitude ratio in both groups was not significantly different which implies a more generalized loss in cone driven macular pathways than a specific loss of RGCs. There were no group differences in the pVEP P100 amplitude or peak time suggesting retinal changes may be the more suitable biomarker in PD. OCT findings of thinner retinal nerve fibre layer and foveal thickness compared to reference range correlated with severity of PD. The authors proposed based on receiver operator characteristics that the N95 amplitude was the best classifier for PD severity. Stanzione et al. (1992) explored the effect of dopamine on the PERG by studying the effect of an intramuscular injection containing 100 mg of sulpiride (a selective dopamine receptor-2 antagonist) on high contrast 1 cpd ssPERG and transient PERGs in 19 healthy volunteers. The authors reported a significantly reduced transient P50 amplitude (with normal peak time) and a delay in the peak-to-peak time of the steady state response (with normal amplitude) and concluded this was evidence for dopamine and the D2 receptor involvement in the clinical findings of reduced PERG amplitudes in PD.

The observation of changes in retinal structure in PD has prompted further structure–function association studies using various electrophysiological, psychophysical, and structural measures to search for early biomarkers and disease progression in PD. Some show a correlation between retinal nerve fiber layer thickness and disease severity in PD whilst small PERG amplitude are associated with reduced quality-of-life measures (Garcia-Martin et al., 2014). A small pilot study, of 8 early-stage PD, highlighted the technical challenges of involuntary muscle contraction in PD causing intrusive myogenic artefact in the electrophysiological recording. Palmowski-Wolfe et al. (2006) found no significant differences in the mfERG, an index of cone function in early-stage PD or the mfERG OPs filtered from the signals, but only from 4/8 subjects had an adequate signal to noise ratio, because of the myogenic intrusion. Kaur et al. (2015) explored mfERG with OCT imaging in 20 PD subjects (early to moderate stage). In moderate stage disease mfERG ratio of the central 2^o^ to peripheral amplitudes were reduced and the retinal nerve fiber layer was thin, in keeping with previous findings of worsening electrophysiological signs with disease progression. In addition, Kaur et al. (2015) reported a delay in the pVEP P100 peak time consistent with macular or ganglion cell dysfunction but also unusually an increase in amplitude which they attributed to the use of dopaminergic medications in the study population.

Nowacka et al. (2015) examined the ERG and OCT correlations in 20 recently diagnosed (< 3 years) PD subjects with early to mild severity and 20 age and sex matched controls. The authors found reduced DA and LA b-wave amplitudes. Those subjects with delayed DA OPs also reported difficulties with dark adaptation and contrast sensitivity. The reduced b-waves and OPs support inner retinal dysfunction involving the amacrine cells affecting retinal sensitivity. There appeared to be no significant differences in the generalized cone function as the LA 30 Hz flicker ERGs were normal. Also, there were no significant differences between groups in retinal nerve fiber later thickness indicating that retinal functional changes are likely to precede structural changes in early PD. Mello et al. (2022) also found no OCT evidence of structural retinal changes in a group of 21 mild to moderate stage PD subjects compared to 19 and sex matched controls but reported similar functional differences to Nowacka et al. (2015). The LA3 b-wave amplitude, and sum of the LA OPs were attenuated in the PD medicated group confirming loss of inner retina function. The PhNR measured peak to trough from the b-wave was reduced suggesting some ganglion cell dysfunction. Given the normal OCT structural measures between groups, imaging may not be a useful early biomarker for PD. For a recent review on structural and functional retinal changes in PD see Alves et al. (2023).

With respect to the EOG findings in PD, Economou and Stefanis (1978) first reported a reduced LP:DTratio in in 20 PD subjects that the authors suggested may be due to reduced melatonin in the RPE given the RPE’s role in generating the light-rise. In a longitudinal study of 5 PD subjects, Ikeda et al. (1994) showed the reduced LP:DTratio was present in stage 2 of the disease but the time to the light peak was significantly delayed in stage 1 and worsened by stage 2 which suggests the LP:DTratio or time to peak of the light rise are affected in line with disease progression. In contrast Palmowski-Wolfe et al. (2006) did not find a significant difference in the EOG or DA and LA ERGs in 8 subects with early stage 1 or 2 PD suggesting that retinal functional changes may not be apparent until disease severity increases. Further larger population studies are needed to assess the clinical use of the EOG in monitoring of disease progression in PD, but given the EOG test duration of 30 min, the shorter 15 min the PERG may be more practical and easier for the patient as the EOG requires the subject to perform steady saccades repeatedly over time.

In summary, the PERG P50 and N95 amplitudes may serve as the best early change of visual dysfunction in PD. There is support for PERGs to mid contrast, mid spatial frequency patterns being best suited to identify early changes (Peppe et al., 1995, 1998) or blue-yellow patterns to elicit a response from the kniocellular pathway (Sartucci et al., 2003). The mfERG, though a test of central retinal function may suffer from low signal to noise ratio associated with tremor and difficult with fixation in a clinical population. Full field ERGs suggest that functional loss in the OPs also may reveal early changes in dopaminergic signaling that are associated with reduced contrast sensitivity early in the disorder (Nowacka et al., 2015). Structural changes with OCT, though reported, are not consistently found in early stages of PD and may not be as helpful as visual electrophysiology for early disease detection and monitoring.

### Mouse models

A complete picture of every animal model that has supported our understanding of disorders affecting the CNS is beyond the scope of this review. Indeed, it could be argued that animal models neither fully replicate the human condition nor experience a disease in the same way as their human counterparts. Here, we take the view that rodent models are designed to specifically mimic an aspect of the disease as opposed to a holistic replication of the human physiological response or experience. With this in mind, we have focused on some of the mouse models that have been used to support the clinical findings in human studies and may help further our understanding of the underlying changes in the structure and function of the retina. For detailed reviews on mouse models and the ERG see McCall and Gregg (2008) and Pardue and Peachey (2014).

### Childhood onset models

ASD mouse models have shown altered retinal structure and function. The neuroligin-4 knock out (NL4-KO) reduces glycine receptor clusters (GlyRα1) clusters in the inner plexiform layer and the phenotype exhibits ASD features of fewer social interactions and greater delays in communication between littermates compared with wild type mice (Jamain et al., 2008; Hoon et al., 2011). In another model, the offspring of mice exposed to valproic acid (VPA), to induce poor social abilities and heightened anxiety, had an upregulation of the mGLUR5 receptor. These mice showed reductions only in the DA a-wave amplitude (Guimarães-Souza et al., 2019). Fragile X mental retardation protein (FMRP) has been associated with ASD symptoms (Darnell et al., 2011). Rossignol et al. (2014) used the FMRP knock out mouse to explore retinal structural and functional changes associated with this model of ASD. In the DA luminance response series, they found reduced a- and b-wave amplitudes with 37% less rod photosensitive protein (rhodopsin) expressed. Similarly, the Engrailed-2 knockout (EN2-KO) mouse model has reduced DA a- and b-wave amplitudes, though contrary to some human studies shows a normal LA ERG response profile (Zhang et al., 2019). The BTBR inbred mouse strain also shows poor sociability and the repetitive behaviors associated with the ASD phenotype. The BTBR inbred mouse strain exhibits disruption to interhemispheric and cortical connectivity giving rise to ASD behavioral like features (Fenlon et al., 2015). In this model, the DA a-wave amplitude was reduced with an increased summed DA OP amplitude at the highest flash strength only of log 2.86 cd.s.m^−2^. Under LA conditions only the a-wave amplitude was smaller, but not the b-wave amplitude (Cheng et al., 2020a).

The several mouse models used in ASD studies exhibit different ERGs, rather like the mixture of results observed in human studies which reflect the complex pathophysiology and clinical heterogeneity of this condition that changes with development (Pretzsch et al., 2022). The mouse models do however provide a method with which to explore specific mechanisms associated with ASD. For example, synaptic development and communication have been implicated in the pathophysiology of ASD (Bourgeron, 2009), and so Hoon et al. (2011) explored the ERG and retinal and cortical expression differences of neuroligin-4 in the NL4-KO mouse model compared to control mice. The study revealed a loss of DA b-wave amplitudes and OPs like those reported in adults with ASD (Ritvo et al., 1988; Constable et al., 2016) and were likely to be due to a loss of innervation by glycine receptors in bipolar cells as visualized by immunohistochemistry (Hoon et al., 2011).

Glutamate and GABA regulation have been implicated in the pathophysiology of ASD (Siegel-Ramsay et al., 2021). The VPA mouse model reported by Guimarães-Souza et al. (2019) offers a way of studying the ERG with reduced GABA. The VPA mouse model shows up-regulation of the mGluR5 receptor in the inner and outer plexiform layers accompanied by down regulation of the synaptic structural proteins synapsin-1 in the inner plexiform layer, and down regulation of GABA in the inner plexiform and ganglion cell layers. Despite these changes in protein expression, only a reduced a-wave amplitude at higher flash strengths in the DA luminance response series was significantly different between the VPA mouse model and control group. The authors suggest that, given no marked histological differences in the thickness of the outer nuclear layer then the changes in the a-wave may reflect alterations in the cone pathway given that no differences were observed at lower flash strengths. Given the lack of significant differences in the OPs or a full study of the LA ERGs in this mouse model it currently does not fully replicate the findings in human such as a reduced DA b-wave amplitude (Ritvo et al., 1988; Constable et al., 2016). The FMRP knock out mouse model in contrast, exhibits reduction of both DA a- and b-wave amplitudes. Histologically the outer nuclear layer thickness was the same but rhodopsin content was less in the FMRP knock out mice and there were a greater number of immature retinal neurons and disorganization of the photoreceptor discs that likely are responsible for the reduced a-wave amplitudes. No LA ERGs or OP analysis were reported (Rossignol et al., 2014). The FMRP knock out mouse gives a similar DA ERG response to human studies reported in adults (Ritvo et al., 1988; Constable et al., 2016).

Zhang et al. (2019) investigated the EN2-KO mouse model of human ASD. Phenotypically the EN2-KO mouse exhibit less social play and grooming with poorer memory and learning ability (Cheh et al., 2006). The EN2-KO retina shows a decrease in cell specific markers for rhodopsin and bipolar cells, a reduced number of horizontal cells, and an increased number of ganglion cells. The ERG findings were consistent with human studies, with a reduced DA b-wave amplitude (Ritvo et al., 1988; Constable et al., 2016), but the LA luminance response series was normal which agrees with the large adult study by Friedel et al. (2022a), though not Constable et al. (2020). The authors attributed the DA b-wave reduction to a loss of rod function associated with a lack of rhodopsin expression, but the DA a-wave amplitude was significantly reduced at the higher flash strengths. This implies the reduced numbers of horizontal cells may have altered the retinal circuits leading to a greater compensatory inhibition at the stronger flash strengths. The BTBR mouse model of ASD also shows a reduced DA a-wave amplitude and peak time delay at the higher flash strengths. Although DA b-wave amplitudes were normal, a reduced DA OPs amplitude sum was found at the strongest flash of log 3 cd.s.m^−2^ (Cheng et al., 2020a,b). The LA a-wave amplitude was markedly reduced, LA b-wave peak time delayed and LA flicker amplitude reduced which were not observed in one human study (Constable et al., 2016). The authors proposed that the reduced LA a-wave was due to impaired calcium homeostasis in the outer retina. No immunohistochemistry was reported in the study.

Dai et al. (2017) described a potential ADHD mouse model in which a homozygous coding substitution in the dopamine transporter (DAT Ala559Val) results in an increase in dopamine efflux in the extracellular space. Phenotypically, the mice exhibited traits in line with ADHD with elevated locomotion and a blunted response to psychostimulants. Of note the LA ERGs had elevated b-wave amplitudes like those reported by Lee et al. (2022), though only in male mice. Interestingly the female mice LA b-wave amplitudes were not elevated in keeping with the sex differences observed by Dubois et al. (2023) with female ADHD individuals having a trend for lower LA b-wave amplitudes. No significant differences were reported in the DA ERGs in the DAT mouse model and there are to date no human studies exploring the DA ERG in ADHD. Another promising model that may support further studies in the ERG and visual perception in ADHD is the dopamine transporter (DAT^+/−^) mouse (Mereu et al., 2017). The DAT^+/−^mouse exhibits a similar phenotype to ADHD with hyperactivity and inattention with less behavioral differences after treatment with amphetamines.

There are no reports yet of ERG findings in human where there is a co-occurrence of conditions such as ASD and ADHD. One mouse model, which may be helpful in these studies has been described by Gao et al. (2022), with a mixed ASD/ADHD phenotype due to knock-out of Kdm6b a histone transcription factor for neural progenitor cells. The mouse displays reduced sociability and cognitive memory which are associated with ASD but with hyperactivity with longer running distances and less stationary behavior that are traits associated with the hyperactivity of ADHD (Gao et al., 2022).

Though the overall findings of the mouse models of ASD do not concur they do provide additional insights into visual processing in ASD. They provide possible ways to explore the underlying mechanisms of the observed ERG findings in human ASD by enabling immunocytochemical analysis of the location and expression patterns of key components of synaptic structure and function which are not available in human studies. For a recent review on mouse and zebrafish models of ASD and ADHD see Dougnon and Matsui (2022), for animal models in ADHD see Stanford (2022).

### Early adult onset models

Glycogen Synthase Kinase-3 (GSK3) has been posited as a possible explanation for the reduced b-waves in schizophrenia and bipolar disorder because upregulation in mice causes ERG findings that mimic human studies (Lavoie et al., 2014a). GSK3 is a protein kinase that interacts with numerous signaling pathways including those that affect cell fate during embryogenesis and microtubule formation and exists as two isoforms (GSK3α and β; Cohen and Frame, 2001). GSK3 expression may be implicated in memory and cognitive decline in schizophrenia and AD (Albeely et al., 2022). An additional model produced through serene racemase gene knockout (SR^−/−^) first described by Basu et al. (2009) causing hypofunction of the N-methyl-D-aspartate acid receptor (NMDAR) has been used as a model for schizophrenia. The model exhibits reduced DA a- and b-wave amplitudes and longer b-wave peak time affecting male more than female mice (Torres Jimenez et al., 2020).

These mouse models for schizophrenia replicate some of the findings observed in human studies. The findings with modulation of GSK3β expression and down regulation of the GSK3α isoforms led to different effects on the recorded mouse ERGs. When GSK3β was over expressed in cortex and retina there was a reduction in the rod driven b-wave and DA luminance response function V_max_ but no change in the LA ERGs. These findings were consistent with children at high risk of developing schizophrenia (Hébert et al., 2010) and implicate GSK3β in the pathophysiology of psychiatric disorders. When GSK3β was upregulated, the ERG findings were reversed with an increase in the DA and LA b-waves in the mouse models supporting the role of GSK3β in modulating the ERG b-wave, through a yet undescribed pathway (Lavoie et al., 2014a). In the GRK3α knock-out mice the authors found an increase in the DA and LA b-wave amplitudes at the higher flash strengths used than the wild-type mice and non-significant reduction in the LA a-wave amplitude, reflecting the more significant reductions in the cone driven a-wave amplitude as reported in human studies (Warner et al., 1999; Balogh et al., 2008). Studies to localize the expression of the GSK3 isoforms within the retina may provide further insights into how alterations in GSK3 expression modulate the b-wave amplitude.

The SR^−/−^ mouse model of schizophrenia shows promise for exploring sex differences in neurological disorders as male and female mice exhibited different responses under some test conditions. Whilst no main group differences were observed under DA or LA conditions, when the ERGs were recorded under mesopic conditions the reduced a- and b-wave amplitudes and delay in peak times were consistent with human studies in schizophrenia suggesting that mesopic testing conditions may improve the b-wave amplitude as a stronger biomarker for schizophrenia (Torres Jimenez et al., 2020) as suggested in recent human studies (Hébert et al., 2015; Demmin et al., 2018). For a review om animal models in schizophrenia see Malik et al. (2023).

### Late adult onset models

**Figure 4:**
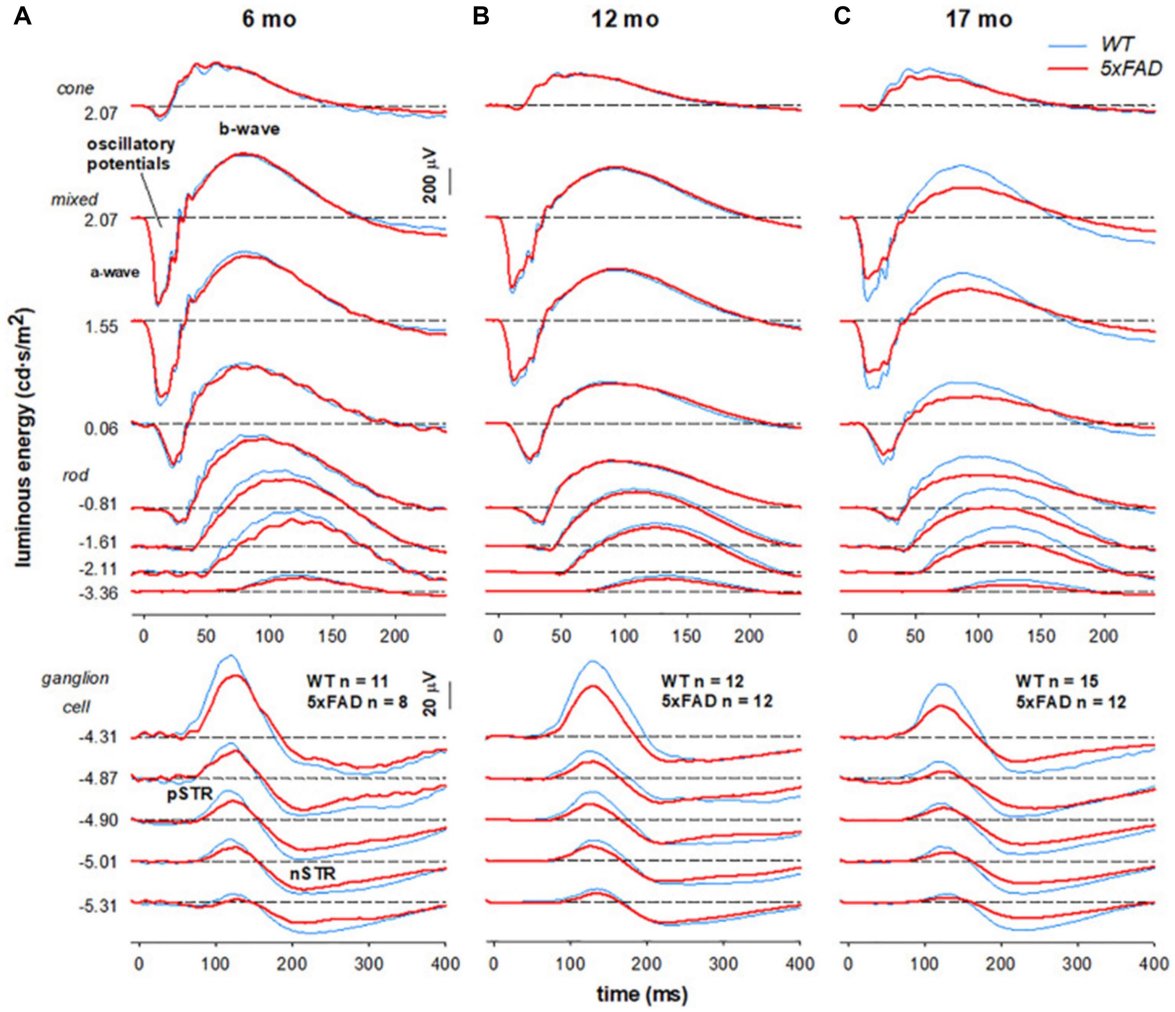
Electroretinography in 5xFAD mice across three ages, showing early changes in the inner retina (ganglion cell function) at 6 months of age, followed by a more generalized loss in retinal function at 17 months of age. The positive (p) and negative (n) Scotopic Threshold Response (STR) indicate reduced ganglion cell function. Note the normal dark adapted full field ERGs in early stages of disease progression at 6 and 12 months in this mouse model. Figure modified from Lim et al. (2020).

For AD, the 5xFAD mouse model has gained traction in recent years due to its ability to replicate major features of human AD in terms of progression, amyloid deposition, frailty, mobility, and dementia (Oblak et al., 2021). The model was originally created by introducing 3 human amyloid precursor protein mutations (APPSwFiLon) and 2 presenilin (PSEN) mutations (M146L, L286V) into the B6SJL mouse model and Thy1 promoter to drive neuronal overexpression. However, mice bred on the B6SJL background carried the retinal degeneration allele Pde6brd1, making the original 5xFAD mouse strain less suitable for visual electrophysiological studies (Oakley et al., 2006). Since 2011, 5xFAD mice produced by the Jackson Laboratory are bred on the C57BL/6J background and backcrossed to remove the retinal degeneration allele Pde6brd1. Even though this model produced less cortical amyloid than its B6SJL predecessor, it did not develop retinal degeneration. In this amyloid only model, it has been demonstrated that the accumulation of amyloid in the retina (Lim et al., 2021) can lead to a reduction of inner retinal function at the early stages, before impacting the outer layers with disease progression (Lim et al., 2020). Notably, a reduction in the RGC function has been recorded as early as 6 months of age as observed by a reduced STR response (Saszik et al., 2002; Bui and Fortune, 2004). The early loss of the STR suggests that post receptoral anomalies are more sensitive to early AD changes (Lim et al., 2020; see Figure 4). Further evidence for RGC dysfunction in the 5xFAD mouse model comes from McAnany et al. (2021) who recorded the PhNR along with the DA and LA luminance response series. The authors reported a delay and reduction in the PhNR and reduced DA b-wave amplitude with normal DA a-waves.

Another mouse model is an amyloid gene knock-in approach, with the App ^NL-G-F^ mouse model used to assist with modelling pre-clinical AD without the potential confounding factor of the prion promoter which leads to over-expression of the amyloid precursor protein (APP). Using this approach, Vandenabeele et al. (2021) showed that the ERG is a sensitive indicator for amyloid toxicity at the pre-plaque stage, with delays in the OPs and a thinning of the outer nuclear layer. One limitation of mouse models is the difficulty in recording PERGs to assess ganglion cell function owing to the small signal to noise ratio compared with full field ERGs and possible loss of media clarity due to oedema or cataract (Porciatti, 2007). However, the 5xFAD mouse model provides the ability to perform imaging and functional tests during disease progression in a homogenous population.

In comparison to human studies the 5xFAD mouse model replicates the key findings observed in human studies of a loss of RGC function (Parisi et al., 2001; Krasodomska et al., 2010; Ngoo et al., 2019; Lim et al., 2020; Asanad et al., 2021; McAnany et al., 2021). Imaging studies using OCT also report a thinning of the retinal nerve fibre layer in the 5xFAD mouse (Lim et al., 2020) model consistent with human studies (Parisi et al., 2001; Moschos et al., 2012). Like human adult studies of the full field ERG, the 5xFAD mouse shows normal DA and LA ERGs in early stages but show reduced inner retinal function in later stages as accumulation of amyloid-β plaques develop with delays in the DA OPs (McAnany et al., 2021; Vandenabeele et al., 2021), DA a-wave peak time (McAnany et al., 2021) and smaller DA b-wave amplitude (Lim et al., 2021; McAnany et al., 2021) with no strong changes in the LA-ERGs. For a recent review on animal models in AD see Chen and Zhang (2022).

**Figure 5:**
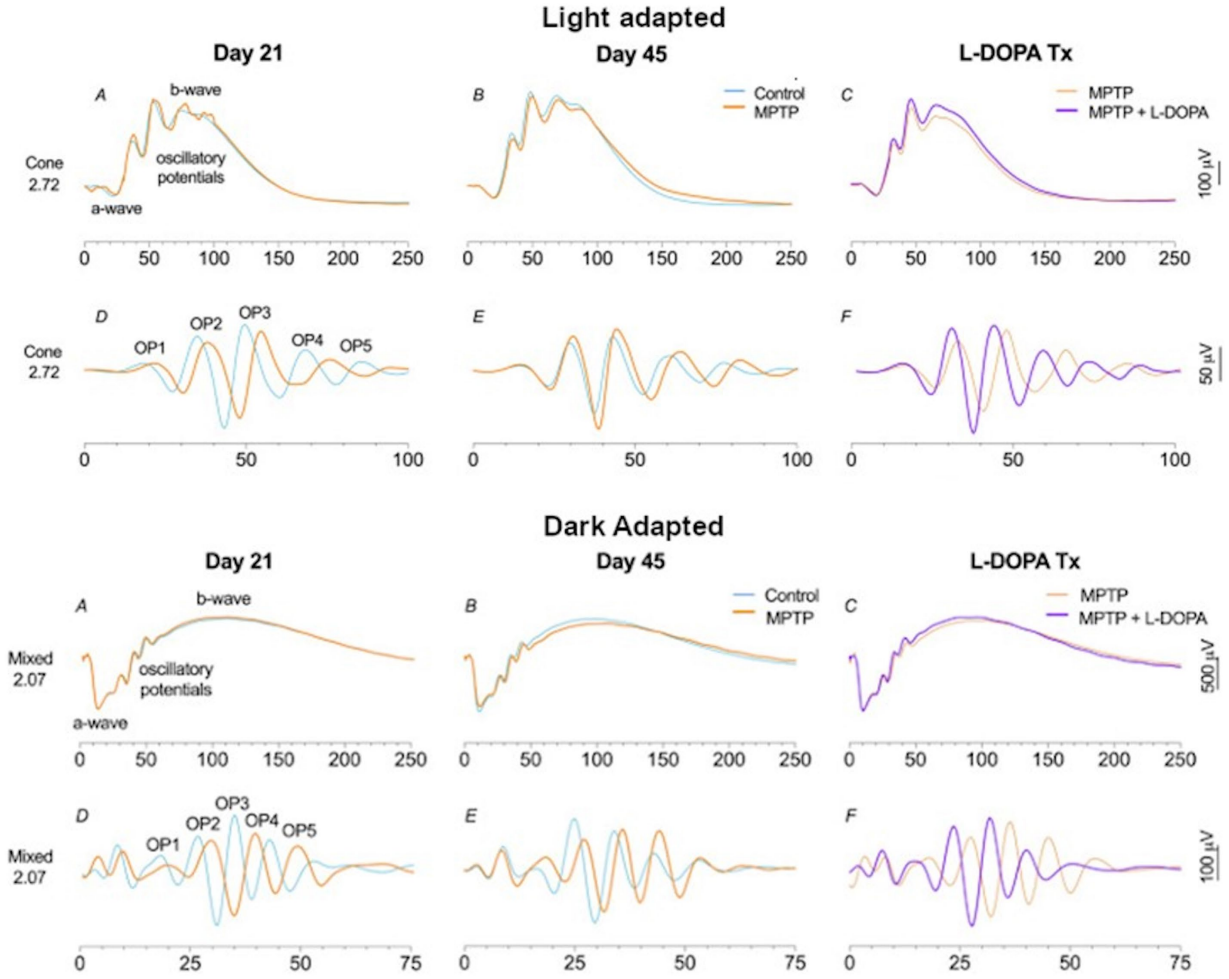
The 1-methyl-4-phenyl-1,2,3,6-tetrahydropyridine (MPTP) toxin induced PD over 21- and 45-days show reduced oscillatory potentials in both the light and dark-adapted ERG. L-dopa treatment shows an amelioration of the OP timing delays in this PD mouse model. Figure modified from Tran et al. (2022).

For PD, the 1-methyl-4-phenyl-1,2,3,6-tetrahydropyridine (MPTP) toxin treated mouse model of PD is one of the most widely used models to assess the effect of drug treatment on the motor and sensory features of human PD (Duty and Jenner, 2011). In the retina, there are dopaminergic cells known as the A18 amacrine cell as well dopaminergic dendrites across the inner and outer retina. Using this model, Tran et al. (2022) showed that DA and LA OPs peak times were selectively delayed after MPTP toxin, but this was ameliorated with L-dopa therapy. All other ERG parameters were unaffected suggesting there was no effect on the neurons of the middle or outer retina. These findings support the idea that the ERG is a useful clinical tool for assessing non-motor function in PD. See Figure 5 for recovery of the DA OPs in the MPTP-mouse model of PD with L-dopamine.

Tran et al. (2023) have also evaluated retinal structure and functional changes in the A53T mouse model of PD. The A53T mouse model results in accumulation of α-synuclein which are intracellular neuronal protein aggregates found in the brains of PD individuals and linked to Lewy body pathophysiology (Goedert et al., 2013). Retinal structural findings using OCT revealed a thinning of the outer plexiform and outer nuclear layers before thinning of the RNFL that occurred later older mice. The LA b-wave amplitude was significantly more reduced in the A53T mouse model than the DA ERGs supporting greater sensitivity for the loss of cone driven pathways which is consistent with full field ERG findings in human studies (Gottlob et al., 1987; Nowacka et al., 2015; Mello et al., 2022). The MPTP and A53T mouse models of PD provide an insight into the early retinal changes apparent through structural imaging or visual electrophysiology in this slowly progressive neurodegenerative disorder.

Although mouse models may not always mimic fully the human CNS disorders, they provide valuable support for clinical studies and allow more detailed structural and functional analyses to be performed within a homogeneous population. In conjunction with human studies the mouse and other animal models will contribute to our greater understanding of the clinical relevance of functional and importantly detailed structural changes in the retina that underpin the clinical findings (Peachey and Ball, 2003; McCall and Gregg, 2008; Pardue and Peachey, 2014; Dai et al., 2017; Torres Jimenez et al., 2020; Cheng et al., 2020a; McAnany et al., 2021; Vandenabeele et al., 2021). For further reviews on animal models in PD see Kin et al. (2019).

## Discussion

One aim of this review was to provide an overview of the many studies that have been conducted harnessing the power of the retina as a window to the brain. This field is expanding rapidly along with advances in new modalities of ocular imaging such as hyperspectral (Lemmens et al., 2020; Reshef et al., 2020) and retinal vasculature (Coffey et al., 2021) that can be used in conjunction with functional data to improve the diagnostic accuracy and management of CNS disorders (Appaji et al., 2019a,b; Lai et al., 2020; Gupta et al., 2021; Kashani et al., 2021; Lim et al., 2021; Vandenabeele et al., 2021; Gross et al., 2022; Komatsu et al., 2022; Maziade et al., 2022; Mohammad-Manjur et al., 2022; Peredo et al., 2022). Although some progress has been made in utilizing functional retina measures as a window to the brain (London et al., 2013; Nguyen et al., 2017; Mahroo, 2023) in psychiatric disorders there are challenges to overcome, such as the potential interactions with smoking, systemic disease such as diabetes, co-occurrence of clinical conditions and past drug use, that pose limitations in some cases (Sanchez-Villarejo et al., 2014; Schwitzer et al., 2021). In addition, the common genetic overlap in conditions that may share similar clinical features, can prevent a direct correlation with a specific clinical diagnostic category (Doherty and Owen, 2014; The Brain Consortium et al., 2018; Antshel and Russo, 2019; Shic et al., 2022). One alternative approach to categorizing CNS disorders discussed by Clementz et al. (2016) is to instead consider classification based on biological phenotype or “*biotype*” that includes a biological and phenotypic marker that better stratifies conditions with overlapping phenotypic features. Here the analysis of the retinal functional measures may help identify biotypes within the neurodevelopmental (Dacquino et al., 2015; Barth et al., 2018; Liang et al., 2019, 2020; Fernández-Linsenbarth et al., 2021) and neurodegenerative domains (Wang et al., 2020) that contribute to a diagnostic categorization system which aligns more closely with neurobiology and clinical phenotype (Brucar et al., 2023).

The greater use of the ERG and the application of new protocols and analytical methods (Mahroo, 2023) offers the potential to build on previous studies that have identified promising retinal biomarkers in CNS disorders (Hébert et al., 2015; Demmin et al., 2018; Almonte et al., 2020; Duraković et al., 2020; Hébert et al., 2020; Silverstein and Thompson, 2020; Lee et al., 2022; Mohammad-Manjur et al., 2022; Silverstein et al., 2022; Tursini et al., 2022; Schwitzer et al., 2022b). The combination of ERG functional metrics with other physiological markers such as eye-movements (Shic et al., 2022), pupillary light response (Guath et al., 2022), sleep patterns (Gross et al., 2022) and retinal structural (Parisi et al., 2001; Garcia-Martin et al., 2014; Kaur et al., 2015; Almonte et al., 2020; Duraković et al., 2020; Sen et al., 2020; Sarkar et al., 2021; Silverstein et al., 2021, 2022; Tran et al., 2022) will allow clustering analyses of neurobiological and phenotypic features that may help develop new classification models and a better understanding of disease progress (Liang et al., 2020).

Some limitations still exist in the classification of neurodevelopmental conditions because individuals may meet diagnostic classification for more than one condition such as ASD, schizophrenia, and ADHD (Simonoff et al., 2008; Chisholm et al., 2015; Bougeard et al., 2021). For example, significant differences in the ERGs can be observed between ASD (smaller b-waves) and ADHD (larger b-waves), but there is a lack of data for when ASD and ADHD co-occur. In this instance these different effects may cancel each other. The LA-ERG results in ASD adult populations are not yet consistent (Constable et al., 2016; Friedel et al., 2022a) suggesting retinal development and ASD severity also may be factors that affects the ERG. Furthermore, no studies to date have been performed in children with related neurodevelopmental disorders such as ADHD-inattentive subtype, language disorder, dyslexia or specific learning disorder that may or may not reveal similar differences and so reduce the specificity of the potential ERG biomarkers in ASD or ADHD. Here mouse models can provide an alternative way to explore in more detail the structural and functional changes in ASD and ADHD retina (Dai et al., 2017; Zhang et al., 2019; Cheng et al., 2020a). The classification of schizophrenia and bipolar disorders based on ERG findings is more promising (Lavoie et al., 2014a; Hosak et al., 2018; Hébert et al., 2020; Tursini et al., 2022) and provides future hope that visual electrophysiology will aid in the early diagnosis (Peredo et al., 2020; Gross et al., 2022; Maziade et al., 2022; Moreau et al., 2022; Peredo et al., 2022) and management of these conditions (Silverstein and Thompson, 2020; Schwitzer et al., 2022b). In a similar way that the PERG shows promise as a potential fast and reliable clinical test to assess RGC and central macular function in PD (Garcia-Martin et al., 2014) and AD (Parisi et al., 2001).

### Retinal electrophysiological changes and neurological disorders

**Figure 6:**
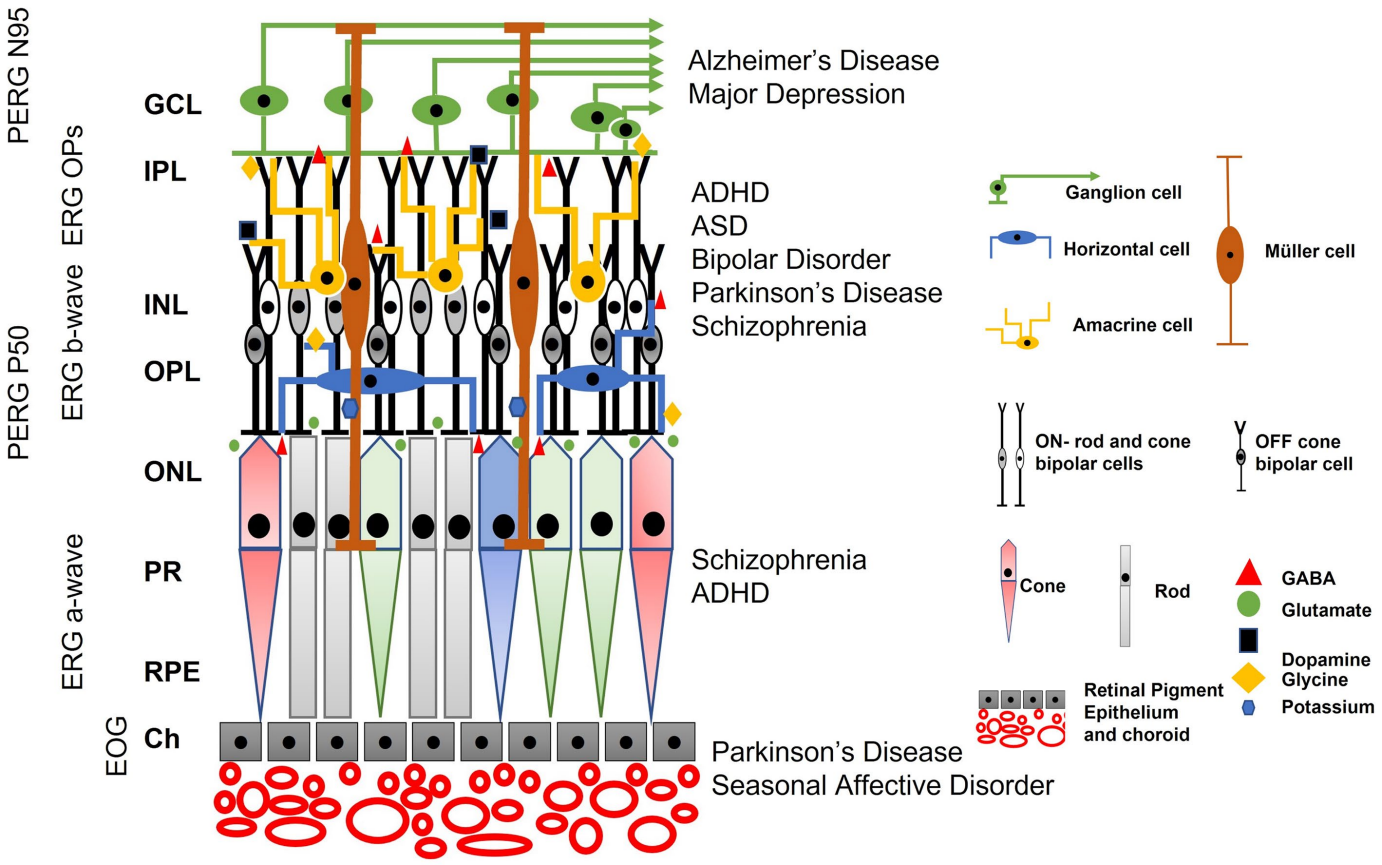
A schematic representation of some of the main functional tests used to evaluate aspects of retinal signaling and their association with neurodevelopmental and degenerative disorders. (Note figure not to scale and not all neurotransmitters are represented such as taurine and acetylcholine). The electro-oculogram (EOG) is formed by the depolarization of the retinal pigment epithelium’s basolateral membrane and has been reported to be reduced in Parkinson’s disease and Seasonal affective disorder. Although dopamine involvement is implicated given its role in the pathophysiology of these conditions, it is uncertain how dopamine may affect the light-rise of the EOG in these conditions. The electroretinogram (ERG) has two principal peaks known as the a-wave that derives largely from hyperpolarization of the outer segments of the photoreceptors and the b-wave that is driven by depolarization of ON- (rods) and ON-and OFF- bipolar cells (cones) that use metabotropic and ionotropic glutamate receptors, respectively. The bipolar and photoreceptors receive inhibitory inputs from horizontal cells (using GABA and glycine) and bipolar cells are modified by amacrine cells that use GABA, dopamine and glycine and thus shape the b-wave. The amacrine cells initiate the high frequency oscillatory potentials (OPs) through inhibitory signaling with bipolar and ganglion cells. The bipolar, horizontal and amacrine cells have all been implicated as possible markers for attention deficit hyperactivity disorder (ADHD), autism spectrum disorder (ASD), schizophrenia, and bipolar disorder based on changes in the ERG and/or OPs waveforms. The PERG has two components: the N95 is defined by retinal ganglion cells (RGCs) and the P50 is driven by cones, bipolar cells and RGCs. Parkinson’s Disease shows loss of the P50 component whilst Alzheimer’s Disease and Depression preferentially affect the RGCs. Key: Ch: choroid, RPE: Retinal Pigment Epithelium, PR: Photoreceptor outer segments, ONL: Outer Nuclear Layer, OPL: Outer Plexiform Layer, INL: Inner Nuclear Layer, IPL, Inner Plexiform layer, GCL: Ganglion cell layer with axons to the lateral geniculate nucleus.

A general framework is presented in Figure 6. This summarizes the main retinal locations, their neurotransmitters and visual electrophysiological tests implicated with the neurological conditions discussed in this review. For additional review on neurotransmitters in the cortex and retina with disease see Nguyen et al. (2017).

Low dopamine is associated with a smaller LA b-wave amplitudes and DA OPs in PD (Nowacka et al., 2015) but a reduced LA b-wave amplitude is not a consistent finding in ASD which suggests interpretation of the ERG as a biomarker for ASD may not be straightforward (Constable et al., 2020, 2022; Lee et al., 2022; Friedel et al., 2022b). Whilst the reduced energy and atypical morphology of the LA OPs in ASD (Constable et al., 2016, 2022) would align with an impaired dopamine signaling in the amacrine cells and may also account for the smaller DA b-wave amplitudes in ASD (Ritvo et al., 1988; Constable et al., 2016), Holopigian et al. (1994) showed in human that there was an effect of specific D1 and D2 receptor blockers (Chlorpromazine and fluphenazine), that reduced the DA b-wave and 30 Hz flicker amplitudes. The DA ERG findings may also implicate dysfunction in glutamate pathways because rod photoreceptors synapse with rod ON bipolar cells that use mGLUR6 to gate open their cation channels before depolarizing to generate the b-wave (Morgans et al., 2009). The ON-pathway deficit reported in ASD based on the extended LA flash ERG (Constable et al., 2016) and mathematical analysis of the LA luminance response function (Constable et al., 2020; Lee et al., 2022) also imply a glutamate signaling abnormality in ASD. Further studies will be required to determine if the ERG findings are relatable to a specific retinal neurotransmitter. For PD, the reduced PERG P50 amplitude and peak time appear to be associated with dysfunction of amacrine cell dopamine signaling pathways because the P50 is lost in healthy subjects who receive a selective dopamine receptor 2 antagonist (Stanzione et al., 1992) as well as attenuation of transient and ssPERGs in clinical PD populations (Calzetti et al., 1990; Tagliati et al., 1996; Garcia-Martin et al., 2014). Reduced LA OPs and LA b-wave amplitudes have also been reported in PD (Mello et al., 2022) that are comparable to the loss of energy observed in the LA OPs in ASD (Constable et al., 2022) and reduced LA b-wave amplitudes (Constable et al., 2016, 2020, 2022; Lee et al., 2022).

The inhibitory neurotransmitter, GABA, is implicated in the pathophysiology of schizophrenia and ERG findings may be related to the genetic and environmental factors that may perturb the GABAergic system (Schmidt and Mirnics, 2015). Increased GABAergic inhibition by horizontal cells that dampens photoreceptor and bipolar cell signals has been proposed in explanation of smaller a- and b-waves in schizophrenia (Hébert et al., 2020). This in part may explain the reduced DA b-wave amplitudes in ASD which is supported by some mouse models, though other mouse models suggest that glutamate is more likely involved (Guimarães-Souza et al., 2019). The SR^−/−^ mouse model of schizophrenia also supports a glutamate dysfunction, associated with reduced expression of N-methyl D-aspartate (NMDA) receptor ionic glutamate receptors (Torres Jimenez et al., 2020).

The elevated b-waves observed in one small ADHD study (Lee et al., 2022) but not another (Dubois et al., 2023) suggest that the hypothsis of a glutamate imbalance with increased excitation in the bipolar cells may not be fully supported despite mutations in glutamate genes implicated in hyperactivity in ADHD (Naaijen et al., 2017). Thus, larger studies in well-defined ADHD clinical populations are required across several age groups to ascertain exactly if there are ERG biomarkers specific for this condition. These studies would be further supported by detailed genetic analyses to help explain any retinal functional differences. Similarly, further studies are required to explore the retinal findings in depression and seasonal affective disorder with the most likely changes linked to dopamine (Hébert et al., 2002). Dopamine is not thought to regulate the light-rise of the EOG directly (Gallemore and Steinberg, 1990; Constable, 2014) which suggests additional factors are involved. Further detailed studies in conditions associated with depression such as post-traumatic stress disorder where visual symptoms exist (Trachtman, 2010) and childhood anxiety (Parsons et al., 2021) may be other areas to explore and determine if the ERG is atypical.

The electrophysiological findings in AD most likely reflect the neurodegenerative nature of this condition with loss of RGC function and consequent PERG and PhNR reductions (Katz et al., 1989; Krasodomska et al., 2010; Mavilio et al., 2020; Asanad et al., 2021), though cone photoreceptor degeneration may contribute to findings of PERG P50 delay (Sen et al., 2020). These functional findings, which are readily recorded, may help monitor the progress, or assess the risk of developing AD along with genetic risk factors (Latimer et al., 2021). Monitoring the magnocellular pathway with specific stimuli may strengthen the potential of visual electrophysiology as an early biomarker (Sartucci et al., 2010). For PD, the PERG at mid spatial frequencies and medium contrast or a blue-yellow koniocellular stimulus may be most useful for the monitoring and early detection of this disorder (Peppe et al., 1995, 1998; Tagliati et al., 1996; Sartucci et al., 2003, 2006). The PERG may be best suited to identifying markers for major depressive disorder or seasonal affective disorder and may also be linked to abnormal dopamine levels that reduces the PERG amplitude due to decoupling of horizontal cells (Roy and Field, 2019; Friedel et al., 2021).

### Future directions

New insights into novel biomarkers may be provided by the analysis of the ERG waveform in the time-frequency or spectral domain where the ON-and OFF-pathways can be clearly identified (Gauvin et al., 2014, 2015, 2016, 2017) as illustrated in Figure 2. Extracting features from signal analysis in combination with machine learning algorithms can identify neurological conditions such as ASD (Mohammad-Manjur et al., 2022) and depression (Schwitzer et al., 2022a). Machine learning has been used successfully to classify glaucoma in mouse models and in the future may provide a powerful tool to help in classification of psychiatric conditions based on retinal signal analysis (Gajendran et al., 2022). Other areas of signal analysis are still to be explored such as variable frequency complex demodulation that provides a high resolution spectral analysis of waveforms (Wang et al., 2006), or a functional data analytical approach where the ERG waveforms could be analyzed as a series of datapoints to identify differences in location scale or shape of the waveforms (Ramsay, 1982; Ramsay and Dalzell, 1991; Ramsay and Silverman, 2005). These additional mathematical approaches to signal analysis, in combination with phenotypic or other neurobiological markers, may provide the necessary fidelity for more accurate earlier diagnoses and provide more targeted interventions and better prognoses for neurological conditions (Dacquino et al., 2015; Clementz et al., 2016; Barth et al., 2018; Liang et al., 2020; Modrzejewska and Bosy-Gąsior, 2023).

In addition, clinical populations often present with co-occurrence of psychiatric disorders and meet multiple clinical DSM or ICD classifications. This is especially the case for ASD and ADHD where the co-occurrence can be as high as 86% in some clinical populations (Bougeard et al., 2021). Depression and anxiety can also be a common feature of neurological conditions owing to the impact they will have on an individual’s quality of life (Bougeard et al., 2021). Thus identifying “groups” based solely on a single clinical phenotype is not always possible and limits many studies findings to the more typical broader clinical spectrum. To overcome this, classification based on genotype may be possible owing to common variants associate with intellectual disability, ASD, ADHD, bipolar disorder, and schizophrenia (Morris-Rosendahl and Crocq, 2020). Future studies will be required to determine if there are common genetic variants that associate with differences in the visual electrophysiological features that may help identify individuals at risk of developing a particular disorder. The size and selection of control reference groups is particularly important to account for the known changes in ERG associated with increasing age (Birch and Anderson, 1992; Kergoat et al., 2002; Neveu et al., 2011), ocular pigment (Wali and Leguire, 1993) and inter-test variance (Grover et al., 2003) to provide robust clinical decision limits. Familial studies suggest that there may be differences in children at risk or within families (Realmuto et al., 1989; Hébert et al., 2010) with affected children that will require larger studies to translate these early observations into a potential clinical test to identify individuals with the greatest likelihood of developing a neurological disorder (Peredo et al., 2020; Maziade et al., 2022; Peredo et al., 2022). Finally, sex differences have been reported in the ERG responses between male and female subjects in ADHD (Dubois et al., 2023) and seasonal affective disorder (Hébert et al., 2004). Thus, there is a need to delineate the influence of sex on the ERG findings given reports of 30% smaller ERGs in males (Brûlé et al., 2007) and differences in hormonal influences on CNS development between males and females (Marrocco and McEwen, 2016).

In conclusion, the application of visual electrophysiology to assist with classification and understanding the underlying neurobiology of psychiatric conditions is a promising new field (Silverstein and Thompson, 2020; Diamond et al., 2022; Silverstein et al., 2022; Mahroo, 2023). The limitations are because of the large heterogeneity in these clinical populations who have common and overlapping phenotypes and genotypes. Large samples are required to stratify the groups clearly (Doherty and Owen, 2014; The Brain Consortium et al., 2018). Further work is required in neurodevelopmental disorders related to language disorders and in cases of co-occurrence of multiple diagnostic conditions to ascertain their influence, if any, on the main findings reported so far in clinical populations that have tried to exclude the co-occurrence of conditions. Further complications arise with the interactions of medications and their potential influence on the electrophysiological findings. Nevertheless, there is a clear potential to expand the clinical utility of visual electrophysiology with neurology. Visual electrophysiology may provide the extra dimension that will complement other biomarker discoveries to aid in earlier diagnoses and management of psychiatric conditions. This review provides a general overview of this developing area that will continue to evolve as more studies in larger and more diverse clinical populations are examined that could extend the clinical application of the ERG and its analysis in neurological disorders (Hamilton, 2021; Thompson, 2021; Mahroo, 2023).

## Author contributions

PC and DT conceived the article. PC and JL wrote the first draft. All authors contributed to the article and approved the submitted version.

## Conflict of interest

The authors declare that the research was conducted in the absence of any commercial or financial relationships that could be construed as a potential conflict of interest.

## Publisher’s note

All claims expressed in this article are solely those of the authors and do not necessarily represent those of their affiliated organizations, or those of the publisher, the editors and the reviewers. Any product that may be evaluated in this article, or claim that may be made by its manufacturer, is not guaranteed or endorsed by the publisher.
